# A Novel Glucocorticoid and Androgen Receptor Modulator Reduces Viral Entry and Innate Immune Inflammatory Responses in the Syrian Hamster Model of SARS-CoV-2 Infection

**DOI:** 10.3389/fimmu.2022.811430

**Published:** 2022-02-16

**Authors:** Savannah M. Rocha, Anna C. Fagre, Amanda S. Latham, Jason E. Cummings, Tawfik A. Aboellail, Philip Reigan, Devin A. Aldaz, Casey P. McDermott, Katriana A. Popichak, Rebekah C. Kading, Tony Schountz, Neil D. Theise, Richard A. Slayden, Ronald B. Tjalkens

**Affiliations:** ^1^Department of Microbiology, Immunology and Pathology, Colorado State University, Fort Collins, CO, United States; ^2^Department of Environmental and Radiological Health Sciences, Colorado State University, Fort Collins, CO, United States; ^3^Skaggs School of Pharmacy and Pharmaceutical Sciences, University of Colorado, Denver, CO, United States; ^4^Depatment of Pathology, New York University (NYU)-Grossman School of Medicine, New York, NY, United States

**Keywords:** COVID-19, SARS – CoV – 2, glucocorticoid receptor, androgen receptor, innate immunity, inflammation, therapeutic, treatment

## Abstract

Despite significant research efforts, treatment options for severe acute respiratory syndrome coronavirus 2 (SARS-CoV-2) remain limited. This is due in part to a lack of therapeutics that increase host defense to the virus. Replication of SARS-CoV-2 in lung tissue is associated with marked infiltration of macrophages and activation of innate immune inflammatory responses that amplify tissue injury. Antagonists of the androgen (AR) and glucocorticoid (GR) receptors have shown efficacy in models of COVID-19 and in clinical studies because the cell surface proteins required for viral entry, angiotensin converting enzyme 2 (ACE2) and the transmembrane protease, serine 2 (TMPRSS2), are transcriptionally regulated by these receptors. We postulated that the GR and AR modulator, PT150, would reduce infectivity of SARS-CoV-2 and prevent inflammatory lung injury in the Syrian golden hamster model of COVID-19 by down-regulating expression of critical genes regulated through these receptors. Animals were infected intranasally with 2.5 × 10^4^ TCID_50_/ml equivalents of SARS-CoV-2 (strain 2019-nCoV/USA-WA1/2020) and PT150 was administered by oral gavage at 30 and 100 mg/Kg/day for a total of 7 days. Animals were examined at 3, 5 and 7 days post-infection (DPI) for lung histopathology, viral load and production of proteins regulating the progression of SARS-CoV-2 infection. Results indicated that oral administration of PT150 caused a dose-dependent decrease in replication of SARS-CoV-2 in lung, as well as in expression of ACE2 and TMPRSS2. Lung hypercellularity and infiltration of macrophages and CD4^+^ T-cells were dramatically decreased in PT150-treated animals, as was tissue damage and expression of IL-6. Molecular docking studies suggest that PT150 binds to the co-activator interface of the ligand-binding domain of both AR and GR, thereby acting as an allosteric modulator and transcriptional repressor of these receptors. Phylogenetic analysis of AR and GR revealed a high degree of sequence identity maintained across multiple species, including humans, suggesting that the mechanism of action and therapeutic efficacy observed in Syrian hamsters would likely be predictive of positive outcomes in patients. PT150 is therefore a strong candidate for further clinical development for the treatment of COVID-19 across variants of SARS-CoV-2.

## Introduction

Following the emergence of a number of idiopathic cases of severe pneumonia in December 2019 in Wuhan, China, deep sequencing of lower respiratory samples from these patients revealed a novel beta-coronavirus that was identified as the causative agent of COVID-19 (1); Zhu et al. (2). As of mid-2021, severe acute respiratory syndrome coronavirus 2 (SARS-CoV-2) has infected over 232 million people and has been responsible for over 5 million deaths (3) and continues to surge in many countries. Transmission of the virus is ongoing globally, and epidemics linked to emerging variants are increasingly detected in many countries (4). The virus can be transmitted through respiratory droplets from asymptomatic, pre-symptomatic and symptomatic carriers (5), making diagnosis and quarantine efforts difficult and ultimately leading to increased propagation and dissemination of infectious virus.

Coronaviruses (Order: *Nidovirales*; Family: *Coronaviridae*) are enveloped, non-segmented, positive-sense RNA viruses that contain very large genomes up to 33.5 kilobases (kb). The four genera of these viruses – *Alphacoronavirus*, *Beta-coronavirus*, *Gamma-coronavirus*, and *Delta-coronavirus* – share a highly conserved genome organization comprising a large replicase gene followed by structural and accessory genes. The organization of the SARS-CoV-2 coronavirus genome is arranged from the 5’-leader-UTR, replicase, S (spike), E (envelope), M (membrane), N (nucleocapsid) to the 3’ UTR poly (A) tail (6). Notably, production of the spike protein has been linked to the severity of the disease. The spike protein is a surface glycoprotein that mediates virus-cell membrane fusion through interaction with the ACE2 receptor and subsequent proteolytic cleavage by TMRPSS2 (7). The spike protein has two domains, the S1 domain comprising residues 12 – 667 and the S2 domain, comprising residues 668 -1273. The S1 subunit contains the receptor-binding domain (RBD), which interacts with the ACE2 receptor, whereas the S2 subunit remains associated with the viral envelope (8). Viral infection requires proteolytic cleavage at Arg685-Ser686 at the S1 site by the transmembrane protease, serine 2 (TMPRSS2), followed by cleavage at the S2 site at Arg815-Ser816 (7). Proteolytic cleavage of the spike protein then enables membrane fusion and entry into the host cell in complex with the ACE2 receptor (9).

Targeting ACE2 and TMPRSS2 has therefore emerged as an important therapeutic strategy for the treatment of COVID-19 by preventing entry of SARS-CoV-2 into cells, thereby limiting viral replication. Both ACE2 and TMPRSS2 are highly expressed in bronchiolar epithelial cells and are transcriptionally regulated by the androgen receptor (AR) through 5’-flanking promoter elements (9). Clinical evidence supports this hypothesis, whereby one prospective study reported a decrease in the rate of intensive care unit admissions in men who had been prescribed anti-androgens for six months prior to hospitalization (10). In another study using a model of SARS-CoV-2 infection, inhibitors of AR transcriptional activity markedly reduced viral infectivity and decreased inflammatory lung injury in experimental animals (9). Both ACE2 and TMPRSS2 are also significantly regulated by inflammation. ACE2, as well as the inflammatory cytokine interleukin-6 (IL-6), are non-canonical interferon-stimulated genes (ISGs) that are highly expressed following infection with SARS-CoV-2 (11). Inflammation also directly upregulates expression of TMPRSS2 (12). Steroids have therefore been extensively used to treat COVID-19 patients, albeit with mixed results. Recent clinical evidence does not support corticosteroid treatment for all cases of SARS-CoV-2-related lung injury in hospitalized patients, which was reported to increase the likelihood for use of mechanical ventilation, vasopressors, and renal replacement therapy if administered during the incorrect phase of infection (13). Corticosteroids such as dexamethasone have been shown to increase expression of ACE2 (14, 15), which would enhance viral entry and replication and could therefore worsen infection when administered too early in the course of disease. Dexamethasone also modestly increased the expression of TMPRSS2 in these studies, which could likewise potentially enhance membrane fusion and vesicular uptake of SARS-CoV-2. Neither hydrocortisone nor prednisolone were able to decrease the expression of TMPRSS2 (14). Thus, better therapeutics are needed that can modulate these receptor systems to downregulate the production of proteins critical to viral entry.

However, classical antagonists of glucocorticoid function that compete for interaction at the steroid binding pocket of the receptor ligand binding domain (LBD) could also be problematic, due to excessive blockade of cortisol function. Allosteric modulators of both AR and GR that could dampen transcriptional activation through these receptors would be preferable as a means of downregulating expression of AR- and GR-regulated genes. Ligands that act as allosteric modulators of nuclear receptors tend to favor stabilization of transcriptional co-repressor proteins on chromatin, such as CoREST, HDAC2/3/4 and NCoR2, that prevent binding of co-activator proteins in response to activation of *cis*-acting transcription factors (16, 17). Thus, ligands functioning as negative regulators of AR and GR could therefore downregulate expression of key genes linked to SARS-CoV-2 infection, including TMPRSS2 and ACE2, as well as numerous inflammatory factors. Currently approved drugs for COVID-19 do not directly address these key host factors of limiting both the replication of SARS-CoV-2 and the ensuing inflammation triggered by infection. To address the potential for a host transcriptional modulator to protect against SARS-CoV-2 infection through downregulation of AR/GR-dependent expression of ACE2 and TMPRS22, we examined the therapeutic efficacy of (11β,17β)-11-(1,3-benzodioxol-5-yl)-17-hydroxy-17-(1-propynyl)-estra-4,9-dien-3-one (designated as “PT150”), a synthetic modulator of GR (18, 19) that we show also decreases expression of AR-regulated genes. In a screening study conducted by the National Institute of Allergy and Infectious Diseases (NIAID), PT150 had broad inhibitory activity towards several RNA viruses, including influenza viruses, Zika virus and beta-coronaviruses, and was recently shown to have direct anti-viral effects against SARS-CoV-2 in human bronchiolar epithelial cells *in vitro* (20). Based on these data, and on previous host-pathogen interaction studies, we postulated that PT150 would be an effective inhibitor of SARS-CoV-2 infection *in vivo* in the Syrian golden hamster model of COVID-19. The results of this study demonstrate that PT150 given orally once daily for 7 days reduced replication of SARS-CoV-2 in lung, decreased infiltration of macrophages, improved lung pathology and reduced expression of both ACE2 and TMPRSS2.

## Materials And Methods

### Virus and Cell Culture Procedures

SARS-CoV-2 strain 2019-nCoV/USA-WA1/2020 was obtained from BEI Resources and propagated on Vero cells (ATCC CCL-81, American Type Culture Collection) at 37°C with 5% CO_2_. Virus titrations from animal tissues were performed by plaque assay as described previously (21). Plaques were visualized two days post inoculation following fixation with 10% formalin for 30 minutes, and addition of 0.25% crystal violet solution in 20% ethanol. All work using infectious virus in animals and in non-fixed cells and tissues was performed in a biosafety level-3 (BSL-3) containment laboratory at the Infectious Disease Research Complex of Colorado State University.

### Animal Procedures

All animal protocols were approved by the Institutional Animal Care and Use Committee at Colorado State University (IACUC Protocol No. 996). Hamsters were used in compliance with the PHS Policy and Guide for the Care and Use of Laboratory Animals and procedures were performed in accordance with National Institutes of Health guidelines. Male and female Syrian hamsters were divided equally and randomly assigned to treatment groups at 8 weeks of age (N=60, Charles River Laboratory). The animals were housed in the CSU animal facility and allowed access to standard pelleted feed and water *ad libitum* prior to being moved to the BSL-3 containment facility for experimental infection. Hamsters were anesthetized by inhalation with isoflurane and then intranasally inoculated with 2.5 × 10^4^ TCID_50_/ml equivalents of SARS-CoV-2 in sterile Dulbecco’s modified Eagles medium (DMEM). Hamsters not receiving SARS-CoV-2 were given a sham inoculation with the equivalent volume of DMEM vehicle. To assess activity of PT150 (supplied by Palisades Therapeutics/Pop Test Oncology LLC) against SARS-CoV-2, experimental groups (*N*=6 animals per group at each timepoint) were as follows: control (sham inoculation + miglyol vehicle), SARS-CoV-2 + miglyol, SARS-CoV-2 + 30 mg/Kg PT150, SARS-CoV-2 + 100 mg/Kg PT150. The experimental drug (PT150) was dissolved in 100% miglyol 812 and delivered by oral gavage at 8μL/g body weight under isoflurane anesthesia. Animals were weighed daily to deliver an accurate dose of drug and were monitored for clinical severity of disease through daily health checks according to an approved clinical scoring matrixs. The SARS-CoV-2 + vehicle group also received miglyol 812 by oral gavage. Animals were observed for clinical signs of disease at time of dosing each day (lethargy, ruffled fur, hunched back posture, nasolacrimal discharge, and rapid breathing). Groups of animals were euthanized at 3, 5 and 7 days post-infection (DPI). Eighteen hamsters (6 SARS-CoV-2 + vehicle, 6 SARS-CoV-2 + 30 mg/Kg PT150; 6 SARS-CoV-2 + 100 mg/Kg PT150) were euthanized at 3 and 5 DPI. On day 7 post-infection (7 DPI) the remaining 24 hamsters were euthanized (6 DMEM + miglyol; 6 SARS-CoV-2 + vehicle, 6 SARS-CoV-2 + 30 mg/Kg PT150; 6 SARS-CoV-2 + 100 mg/Kg PT150). Animals were euthanized by decapitation under isoflurane anesthesia and tissue was collected for immunohistochemistry, viral isolation, RNA analysis and histopathology.

### SARS-CoV-2 RNA Isolation and Determination of Genome Copy Number

The 2019-nCoV CDC qPCR Probe Assay, which targets regions within the nCoV nucleocapsid gene, was used and adapted here. In brief, RNA was isolated from hamster lung by homogenizing tissue in trizol reagent using 5mm stainless steel beads and Qiagen TissueLyser II homogenizer under BSL-3 conditions. RNA was then extracted from individual samples using previously described methods (22). RNA purity and concentration was confirmed using a NanoDrop ND-1000 spectrophotometer (NanoDrop Technologies, Wilmington, DE). Twenty-five nanograms of total RNA was loaded into a 20 μl reaction in which the RNA was reverse transcribed and amplified in a one-step reaction using the Fast 1-Step Mix Taqman (Applied Biosystems, Waltham, MA). Primers and probe were designed and purchased *via* Integrated DNA Technologies (IDT; Coralville, IA): forward primer nCOV_N1(5’-GACCCCAAAATCAGCGAAAT-3’; Cat 10006821; Lot 0000571823), probe nCOV_N1 (5’- FAM-ACCCCGCATTACGTTTGGTGGACC-BHQ1-3’; Cat 10006823; Lot 0000561844), and reverse primer nCOV_N1 (5’-TCTGGTTACTGCCAGTTGAATCTG-3’; Cat 10006822; Lot 0000569191) (23, 24). Each qRT-PCR Assay contained 0.4 μM forward and reverse primers, 0.2 μM probe and 5 μl Fast 1-Step Mix Taqman. RT-PCR conditions were as follows: an initial reverse transcription for 5 min at 50°C followed by a PCR inactivation/initial denaturation for 20 sec at 95°C, and 40 cycles of amplification (3 sec at 95°C, 30 seconds at 60°C). To calculate viral genome copies, a 10-fold serial dilution standard curve was generated concurrently using the standard 2019-nCoV Positive Control Plasmid (IDT; Cat 10006625; Lot 0000568956; delivered at 250 ul, 200,000 copies/ul), which contains the complete nucleocapsid gene from 2019-nCoV. Total viral copy number was calculated and quantified based off standard curve (r^2^ = 0.991) equation generated.

### Histopathology

Lungs from 60 hamsters were extirpated *en bloc* and fixed whole in 10% neutral buffered formalin under BSL-3 containment for at least 72 hours before being transferred to CSU Veterinary Diagnostic Laboratory, BSL-2 necropsy area for tissue trimming and sectioning. Four transverse whole-lung sections were stained with hematoxylin and eosin (H&E). Tissue was sectioned at 5µm thickness and were mounted onto poly-ionic slides. Sections were then deparaffinized and immunostained using the Leica Bond RX_m_ automated robotic staining system. Antigen retrieval was performed by using Bond Epitope Retrieval Solution 1 for 20 minutes in conjunction with base plate heat application. Sections were then permeabilized (0.1% Triton X in 1X TBS) and blocked with 1% donkey serum. Primary antibodies were diluted to their optimized dilutions in tris-buffered saline and incubated on the tissue for 1 hour/antibody: Rabbit SARS nucleocapsid protein (Rockland Cat# 200-401-A50, RRID : AB_828403), goat ionized calcium binding adaptor molecule 1 (Abcam Cat# ab5076, RRID : AB_2224402), goat angiotensin converting enzyme 2 (R and D Systems Cat# AF933, RRID : AB_355722), rabbit transmembrane serine protease 2 (Abcam Cat# ab56111, RRID : AB_883079), mouse interleukin 6 (ThermoFisher Cat# M620, RRID : AB_223576). Sections were then stained for DAPI (ThermoFisher) and were mounted on glass coverslips using ProLong Gold Anti-Fade medium and stored at 4°C until imaging.

### Immunofluorescence Imaging and Protein Quantification

The studies described here were conducted by a single investigator. Images were captured using an automated stage Olympus BX63 fluorescent microscope equipped with a Hamamatsu ORCA-flash 4.0 LT CCD camera and collected using Olympus CellSens software. Quantification of protein was performed by acquiring five randomized images encompassing the pseudostratified columnar epithelium around bronchi at 400x magnification (Olympus X-Apochromat air objective; N.A. 0.95) all from different lung lobes. Regions of interest were then drawn to enclose the epithelial layer and exclude the lumen, in order to accurately obtain average intensity measurements. The Count and Measure function on Olympus CellSens software was then used to threshold the entirety of the ROI and measure the given channel signal. Quantification of invading inflammatory cells was performed by generating whole lung montages by compiling 100x images acquired using automated stage coordinate mapping with an Olympus 10X air objective (0.40 N.A.) All images were obtained and analyzed under the same conditions for magnification, exposure time, lamp intensity, camera gain, and filter application. ROIs were drawn around the lung sections and the co-localization function of Count and Measure within Olympus CellSens software was applied the sections. IBA1^+^ cells were determined per 1 mm^2^ areas given the previously drawn ROI overall area.

### Quantification of Inflammatory Cell Infiltration and Consolidation

Quantification of the total affected pulmonary parenchyma as well as counting of inflammatory cells per area (region of interest, ROI, 1mm^2^) was determined in hematoxylin and eosin (H&E) -stained histological sections by digital image analysis. A digital montage was compiled at 100X magnification using an Olympus X-Apochromat 10X air objective (N.A. 0.40) consisting of approximately 1,200 individual frames per lung lobe. Affected regions of interest (ROI) were subsequently automatically identified using Olympus CellSens software by quantifying whole-lung montages scanned from each hamster for total number of nuclei or nucleated cells (to exclude erythrocytes) stained with H&E, relative to the total area of the ROI for each lung.

### Computational-Based Modeling

PT150 was docked into the crystal structures of the ligand-binding domain of the androgen receptor (PDB: 2PIT) (25), and the glucocorticoid receptor (PDB: 3CLD) (26), using the Glide module within Schrödinger (Release 2020-2, Schrödinger LLC, New York, NY) (27–29). Prior to docking, the water molecules were removed, and the proteins were prepared by assigning bond orders, adding hydrogens, and repairing any side chains or missing amino acid sequences. To complete protein preparation a restrained minimization of the protein structure was performed using the default constraint of 0.30Å RMSD and the OPLS_2005 force field (30). The prepared proteins were subjected to SiteMap analysis (29), which identified the available binding sites in the ligand binding domains of the androgen and glucocorticoid receptors and docking grids were generated using Receptor Grid Generation. PT150 prepared using LigPrep by generating possible states at the target pH 7.0 using Epik and minimized by applying the OPLS_2005 force field (30). Molecular docking simulations were performed targeting each potential binding site for PT150 using the Glide ligand docking module in XP (extra precision) mode and included post-docking minimization (28).

### Phylogenetic Analysis and Similarity Score Representation

Phylogenetic analysis was performed by aligning protein coding sequences across multiple species within the protein of interest. FASTA files were downloaded from National Center for Biotechnology Institute’s (NCBI) gene databases and were then input into Molecular Evolutionary Genetic Analysis (MEGAX, v.10.1.8) software for alignment. Muscle alignment was performed, and the evolutionary history was inferred using Neighbor-Joining methodology (31) resulting in the optimal tree. Percentage of replicate trees in which the associated taxa clustered together was determined through bootstrap testing (5000 replicates), of which is shown next to the respective branches (32). The evolutionary distances were then computed using the Poisson correction method (33). Similarity scores between species were generated by utilizing the Basic Local Alignment Search Tool for protein-protein comparison on the National Center for Biotechnology Information interface.

### Quantification of RNA Transcript Levels

Genes involved in the androgen and glucocorticoid receptor pathways were chosen and primer pairs developed having PCR efficiency values of 0.9-1.1. Actb (Beta-actin) was used as the internal standard reference gene. Primer pairs used in qRT-PCR are listed in **Table S1**. cDNA was prepared from total RNA samples using Transcriptor First Strand cDNA Synthesis kit (Roche Applied Science), as well as using previously described methods (34). The resultant cDNA was used in downstream real-time PCR assays on a Roche LightCycler 480. Samples were added to primer, LightCycler480 SYBR green I master mix (Roche), and water to final volume of 20 μl. Real-time PCR cycle parameters were as follows: pre-incubated at 95°C for 5 minutes, followed by 45 cycles of 95°C for 10 sec, 60°C for 10 sec, and 72°C for 10 sec. All biological replicate samples (*n*=6/group) were quantified independently in technical triplicate. The resultant Cp values were used to calculate relative gene expression by use of the Pfaffl method (35). Relative gene expression values were then input into ClustVis multivariate data analysis and visualization platform (36) where heatmap generation was performed by Pareto scaling for gene-gene scaling within infection-treatment groupings, or unit variance scaling for group comparisons within regard to an individual gene. Correlation distance clustering and Ward linkage analysis (37) was performed for gene grouping whereas correlation distance clustering and average linkage analysis was performed for infection-treatment groupings. Correlation clustering was calculated through pairwise distances where objects with the smallest distance were merged in each step. Pearson’s correlation coefficient subtracted from one was then applied to obtain correlational branching.

### Generation of STRING Functional Association Networks

Genes of interest were entered into STRING protein-protein interaction database (V11.5) (37) and viewed as full STRING networks where edges indicate functional and physical protein associations. Network edges were plotted based on confidence of network where the line thickness represents the strength of the supporting data. Active interaction sources included text mining, experimentation, databases, co-expression, neighborhood, gene fusion and co-occurrence, all with a minimum interaction score of 0.70 representative of high confidence.

### Generation of Representative Normalized Pathological Overlays

To model the temporal sequence of each cellular response in SARS-CoV-2-infected hamsters, normalized pathological overlays were generated for each parameter encompassing all time points examined (Day 3, 5 and 7). Parameters modeled included SARS-CoV-2 viral load (based on the total lung area with nucleocapsid protein), the number of CD4^+^ T-cells as a percent of lung area, the overall intensity of IL-6 expression as a percent of lung area, and the overall percent of lung area occupied by IBA1^+^ macrophages. These responses were normalized to control values according to the following equation:


((x−y)/(y−z))×100=normalized percentage value

(38). The control values obtained for each pathological parameter were averaged and then subtracted from the individual experimentally infected animal values yielding a pathological representative of activation (x). The minimum (y) and maximum (z) values were determined for each activation parameter dataset. Normalization was then performed by determining the difference between the control subtracted experimental infected values and the minimum overall value. The total obtained from this calculation serves as the numerator. The range of the data set was then determined by obtaining the difference from the minimum (y) and maximum (z) values, this would serve as the denominator. The value obtained from this overall calculation was then multiplied by 100 to represent total percentage activation of each parameter. The respective percentages at each time point (2, 3 and 4 WPI) were averaged and plotted using spline curve fitting within Prism software (version 9.1.0; GraphPad Software, San Diego, CA).

### Statistical Analysis

All data is presented as mean +/- SEM, unless otherwise noted. Experimental values from each mean were analyzed with a ROUT (α=0.05) test for exclusion of significant outliers. Differences between each experimental group were analyzed using an unpaired t-test or a one-way ANOVA with Tukey’s *post hoc* test for multiple comparisons. Differences between two variables were identified using a two-way ANOVA following a Tukey *post hoc* multiple comparisons test. Significance is denoted as *p < 0.05, **p < 0.01, ***p < 0.001, ****p < 0.0001. All statistical analysis was conducted using Prism.

## Results

### Clinical Observations and Levels of SARS-CoV-2 in the Lungs of Syrian Hamsters Treated With PT150

**Figure 1:**
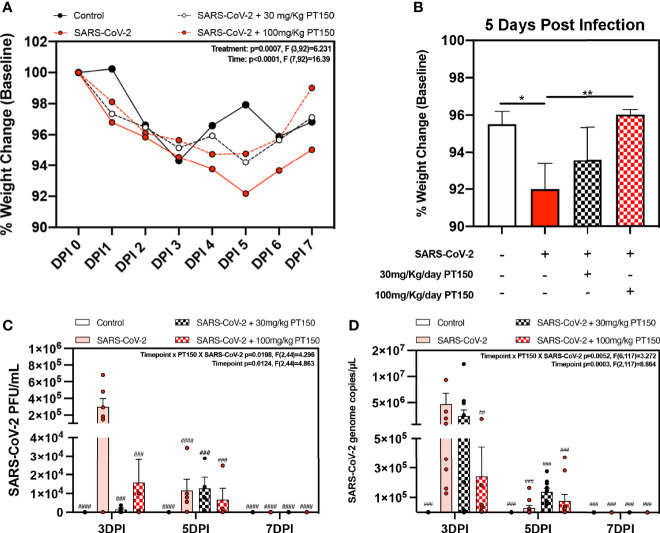
Clinical observations and levels of SARS-CoV-2 in lungs of Syrian hamsters. **(A)** Time course of body weights for all groups plotted as percent change from baseline for each animal. Two-way ANOVA analysis indicated significance for both time (p<0.0001, F (7, 92) = 16.39) and treatment condition (*p*=0.0007, F *3, 92) = 6.231). Maximal loss of body weight was observed by 5 DPI. **(B)** Body weights were directly compared between groups at 5 DPI. Significant differences were detected between the control and SARS-CoV-2 groups, as well as between SARS-CoV-2 + 30 mg/Kg PT150. The SARS-CoV-2 + 100 mg/Kg PT150 group was not different from the SARS-CoV-2 + vehicle group but was also not different from control. **(C, D)** Viral plaquing and transcript analysis of homogenized hamster lung tissue yielding significant viral presence at 3 DPI, where treatment with PT150 showed dose dependent decreases in virus accumulation. Groups were compared by one-way and two-way ANOVA using Tukey’s *post hoc* test; *p<0.05, **p<0.01, ^##^*p*<0.01, ^###^*p*<0.001, ^####^*p*<0.0001 indicating significance from 3 DPI SARS-CoV-2 infected animals; n=6 animals per group.

Oral gavage with PT150 or vehicle began on the same day as infection with 2.5 x 10^4^ TCID_50_ SARS-CoV-2 (USA-WA1-2020 strain) by intranasal inoculation. Body weights were monitored daily for each animal, with a noted decline in average body weight in each experimental group that reached a maximum loss by day 5 with a total overall loss of eight-percent body weight (**Figures 1A, B**). This finding is consistent with other longitudinal studies in Syrian golden hamsters infected with SARS-CoV-2 that demonstrate the maximal clinical severity of disease at day 5 post-infection (39, 40). Infected hamsters treated with vehicle showed the greatest decline in body weight relative to controls, that was prevented by treatment with PT150 at 30 and 100 mg/Kg/day (**Figure 1A**). Two-way ANOVA analysis indicated a difference with treatment (*p*<0.0007, F (3,92)=6.231) and time post-infection (*p*<0.0001, F (7,92)=16.39). Hamsters treated with PT150 at 30 and 100 mg/Kg/day did not show a statistically significant difference in body weight from control hamsters at day 5 post-infection, at which the maximal extent of body weight loss is observed in Syrian hamsters infected with SARS-CoV-2 and treated with vehicle only (**Figure 1B**). Hamsters challenged with SARS-CoV-2 showed marked lethargy, ataxia, ruffled fur, altered posture and overall decreased movement and exploratory behavior, clinical signs and behaviors that were observably less pronounced in hamsters treated with 30 mg/Kg/day PT150. Infected hamsters treated with 100 mg/Kg/day PT150 displayed clinical behavior and body weight changes largely indistinguishable from uninfected control animals (**Figure 1B**). Viral plaquing and genomic copy assessment assays of homogenized hamster lung tissue revealed significant increases in viral titers and RNA copies within the lung at 3 DPI, where both treatment groups (30 mg/kg and 100 mg/kg PT150) showed marked decreases in viral titers compared to the 3 DPI SARS-CoV-2 group (**Figures 1C, D**). The doses of PT150 administered in the *in vivo* studies in hamsters are comparable to calculated human doses. Previous clinical steady-state exposure data in human trials with 500 mg PT150 translates to a dose of approximately 7 mg/Kg. Compared to both rodent and dog, human calculated exposures are significantly greater for a given dose, based on standard physiologic-based pharmacokinetic models that account for differences in mass, body surface area (BSA), metabolism, half-life, etc. (41). Using scaling factors based on BSA, the doses used in the hamster study translate to a Human Equivalent Dose (HED) of: HED = 30 mg/kg x 5/37 = 4.1 mg/kg; HED = 100 mg/kg x 5/37 = 13.5 mg/kg. Thus, 900 mg/day in patients would represent approximately 12.6 mg/Kg, which is very close to the efficacious dose observed in hamsters treated with PT150.

### Lung Histology

**Figure 2:**
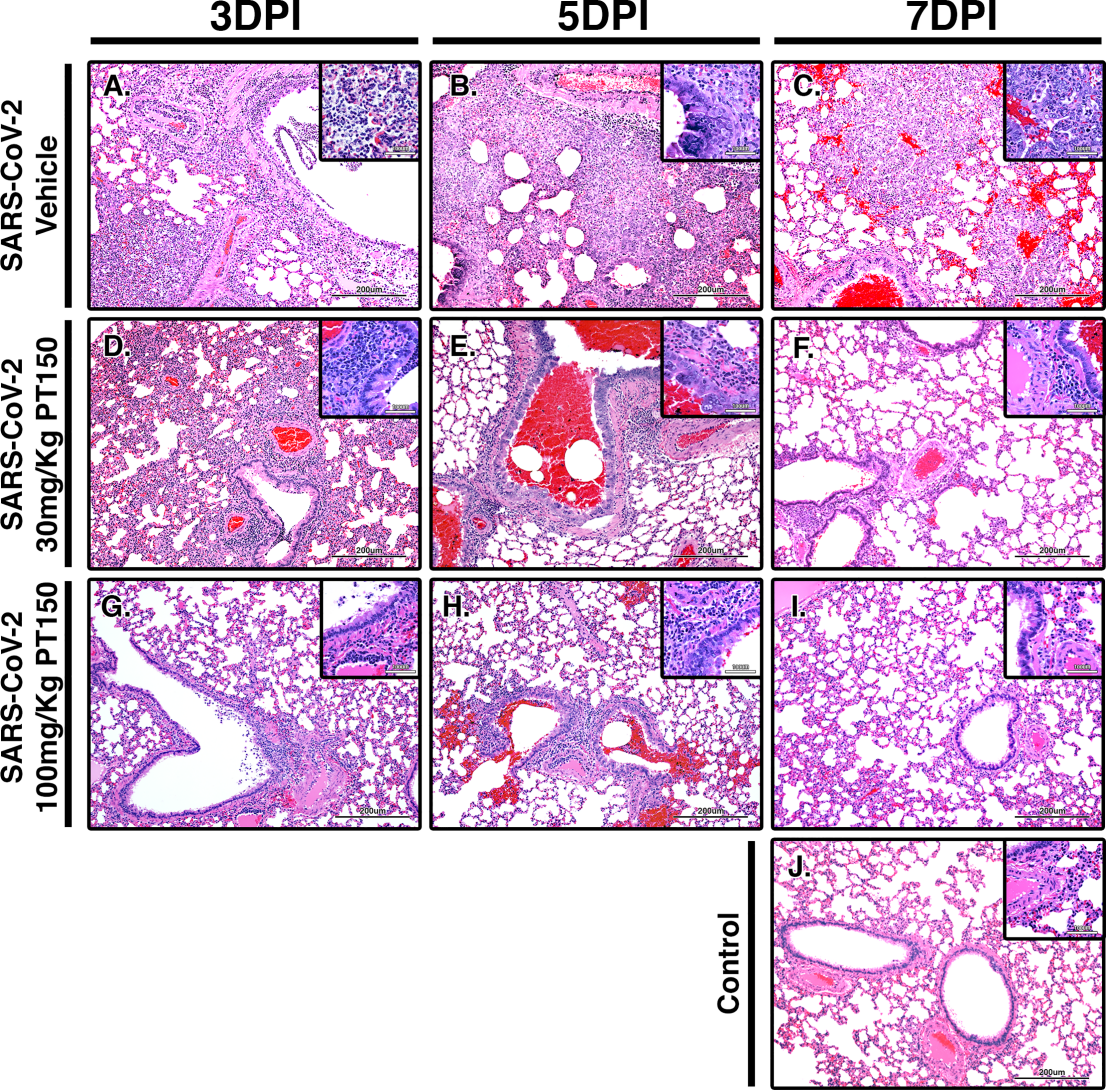
PT150 protects against adverse histopathological outcomes in the lungs of Syrian hamsters infected with SARS-CoV-2. Hamsters were infected by intranasal inoculation with 2.5 × 10^4^ TCID_50_/ml equivalents of SARS-CoV-2 (strain 2019-nCoV/USA-WA1/2020) and lung tissue was stained with hematoxylin and eosin (H&E) for examination of pathological changes on days 3, 5 and 7 post-infection. Treatment groups were **(A–C)** SARS-CoV-2 + vehicle, **(D–F)**, SARS-CoV-2 + 30 mg/Kg/day PT150, **(G–I)** SARS-CoV-2 + 100 mg/Kg/day PT150 and **(J)** Control + vehicle. *N*=6 animals per group. Images were collected at 100X overall magnification (large image panels) or 400X overall magnification (inset panels).

Paraffin-embedded lung sections were stained with hematoxylin and eosin (H&E) and examined by a veterinary pathologist blinded to the treatment groups. Representative lung sections from each experimental group are presented in **Figure 2**. In hamsters infected with SARS-CoV-2, affected portions of lungs showed marked histiocytic/neutrophilic broncho-interstitial pneumonia with hyperplasia of type II pneumocytes and formation of both bronchiolar and alveolar syncytial cells (**Figures 2A**–**C**, low power images and high magnification insets). Inflammation equally involved main branches of the pulmonary artery, where infiltrating macrophages were observed dissecting the tunica media and lifting the tunica intima, with clustering of circulating monocytes along hypertrophied, apoptotic and occasionally hyperplastic endothelial lining cells. Lungs from vehicle-treated infected hamsters show approximately 70% destruction of pulmonary parenchyma by an intense mixed inflammatory infiltrate with a marked expansion of alveolar interstitium, as well as peri-bronchial inflammation and arteritis of large pulmonary vessels. Of note, peri-bronchial inflammation and arteritis of a large pulmonary vessel were observed (**Figure 2B**). High power images (**Figures 2B, C**, insets) of the bronchiolar wall show inflammatory cells comprising macrophages and neutrophils dissecting through bronchiolar muscular wall with similar infiltration of several rows of monocytes/macrophages lifting the tunica intima and dissecting the tunica media. By 7 DPI, infected animals treated with vehicle-only (**Figure 2C**) had less inflammation, with clearing of luminal infiltrates in smaller bronchioles. High power images show reduced clustering of monocytes/macrophages in pulmonary arteries with lingering inflammation in medium-sized bronchioles and parenchymal consolidation. Treatment with PT150 at 30 mg/Kg/day (**Figures 2D**–**F**) and 100 mg/Kg/day (**Figures 2G**–**I**) alleviated the extent of inflammation in main stem bronchi and respiratory bronchioles, as well as in medium-sized pulmonary arteries. The percentage of normal to affected parenchyma showing significant resolution was markedly increased in PT150-treated animals, particularly in the 100 mg/Kg/day group, even at the peak of inflammatory infiltration by 5 DPI (**Figure 2H**, low and high-power images). In the 100 mg/Kg/day treatment group, there was near complete resolution within the parenchyma by 7 DPI (**Figure 2I**, low and high-power images), relative to untreated animals infected with SARS-CoV-2, which showed significant consolidation and loss of parenchymal structure by 7 DPI (**Figure 2C**, low and high-power images). In addition, the number of apoptotic endothelial cells in pulmonary bronchi was greatly reduced by treatment with 100 mg/Kg/day PT150 (**Figures 2G**–**I**, high magnification insets). In hamsters treated with 100 mg/Kg/day PT150, there was a marked reduction in the inflammatory response within bronchioles, interstitium and arteries, with almost complete resolution of bronchiolar inflammation and reduced clustering of circulating monocytes to a marginating single row. No medial dissection is observed in these vessels.

### Analysis of Immune Cell Infiltration and Broncho-Interstitial Pneumonia in Lung Tissue of Animals Exposed to SARS-CoV-2

**Figure 3:**
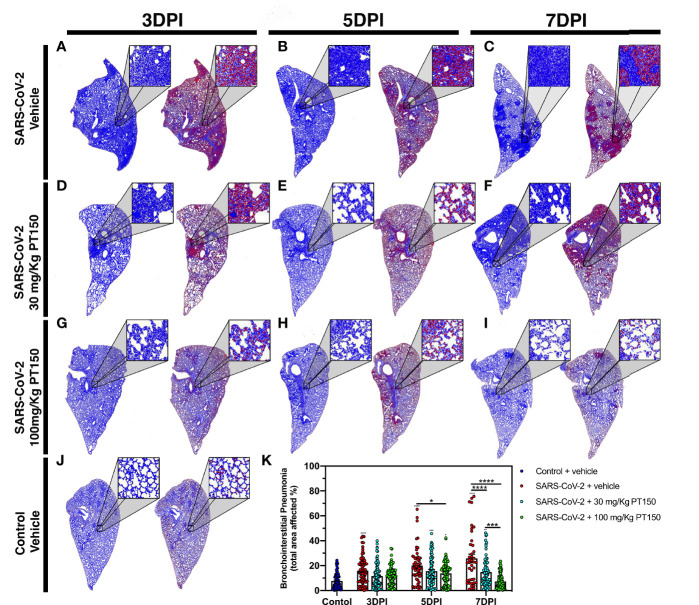
Reduction of immune cell infiltration and broncho-interstitial pneumonia by PT150 treatment. Overall hypercellularity within lung tissue, resulting in pathological broncho-interstitial pneumonia, was determined using ROI delineations on hematoxylin and eosin-stained sections for all groups: days 3, 5 and 7 post-infection. **(A–C)** SARS-CoV-2 + vehicle; **(D–F)**, SARS-CoV-2 + 30 mg/Kg/day PT150; **(G–I)**, SARS-CoV-2 + 100 mg/Kg/day PT150; B, Control + vehicle. **(J)**, Quantification of the total area of the lung tissue affected with broncho-interstitial pneumonia was conducted using automated focal point determination within ROIs following manual thresholding **(K)**. Pseudo colored grayscale images of H&E sections are depicted in blue, overlaid with ROI’s detected by intensity thresholding in red. *p<0.01, ***p<0.001, ****p<0.0001; *N*=6 hamsters/group.

Lungs were examined for the extent of immune cell infiltration at 3, 5 and 7 DPI by quantitative digital image analysis (**Figure 3**). Whole mount sections of paraffin-embedded lung tissue were stained with H&E and bright field grayscale images were collected using a microscope equipped with a scanning motorized stage. Pseudo-colored H&E images are depicted in blue, overlaid with ROIs detected by intensity thresholding in red. Hematoxylin-positive immune cell soma were rendered as focal points within the regions of interest to calculate the percent hypercellularity of tissue following infection with SARS-CoV-2. By 3 DPI, lung tissue showed significant infiltration of immune cells in the SARS-CoV-2 + vehicle group in addition to widespread hemorrhaging (**Figure 3A**). Immune cell infiltration was decreased in dose-dependent fashion by treatment with PT150 at 30 and 100 mg/Kg/day (**Figures 3D**–**I**). Uninfected control hamsters treated with vehicle-only, displayed minimal levels of macrophage hypercellularity with clear bronchi and open parenchyma and an absence of inflammatory infiltrate (**Figure 3J**). The percent of total lung area displaying immune cell hypercellularity was quantified by ROI thresholding and normalizing to the total lung section area (**Figure 3K**). This effectively showed the post-infection response mediated by immune-cell infiltration. There was a time-dependent increase in cellular reactivity, tissue pathology and bronchio-interstitial pneumonia. The peak of cellular infiltration occurred at the 7-day timepoint revealing progressive consolidation. These effects are decreased with the administration of PT150 at the 30 mg/Kg/day dose as well as the 100 mg/Kg/day dose.

### Phylogenetic Analysis and Molecular Docking With the Androgen and Glucocorticoid Receptors

**Figure 4:**
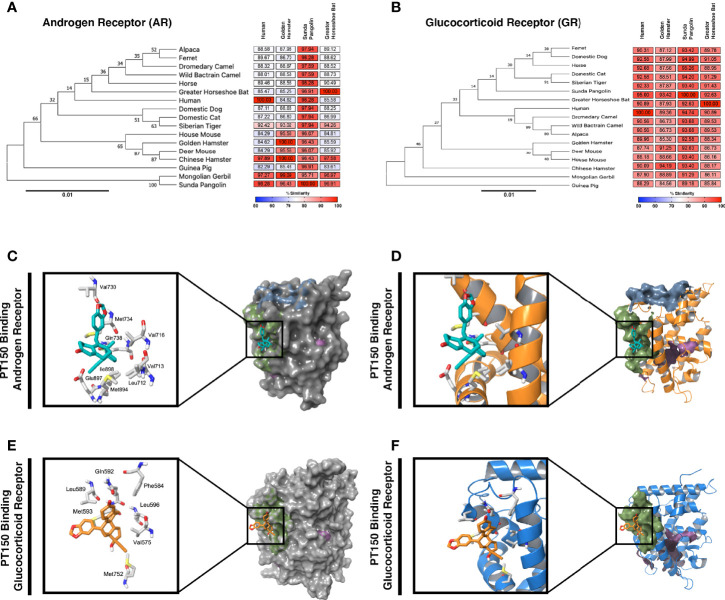
Phylogenetic analysis and molecular docking of PT150 with the androgen and glucocorticoid receptor. Phylogenetic analysis of the androgen receptor **(A)** and glucocorticoid receptor **(B)** indicate a high degree of homology across species, including between human and hamster, indicating that the hamster is an appropriate predictive model to test the protective effects of PT150 against SARS-CoV-2 *via* modulation of these receptors. The putative binding site on the androgen **(C, D)** and glucocorticoid **(E, F)** receptors was analyzed by molecular docking simulations using coordinates from the crystal structures of the human ligand binding domain for AR (PDB: 2PIT) and GR (3CLD) from the Protein Data Bank.

Because expression of TMPRSS2 and ACE2 are regulated through the andro-corticosteroid signaling pathway (8, 12, 42), we examined the genetic sequence identity of the glucocorticoid and androgen receptors across multiple species associated with propagation of SARS-CoV-2 (**Figure 4**). Comparing sequences of the androgen receptor (**Figure 4A**) indicated a high degree of identity between all species analyzed, with sequence concordance values with the human gene ranging from 84.62 (golden hamster) and 85.47 (greater horseshoe bat) to 98.26 (Sunda pangolin). Similarly, sequence concordance values with the human gene for the glucocorticoid receptor (**Figure 4B**) ranged from 89.96 (golden hamster) and 90.89 (greater horseshoe bat) to 95.00 (Sunda pangolin). Sequence similarity between these genes amongst different species is relevant for both potential zoonotic propagation of SARS-CoV-2 as well as to the testing of potential therapeutic compounds acting through the androgen and glucocorticoid receptors.

PT150 was docked into the crystal structures of the ligand-binding domain of the androgen receptor (PDB: 2PIT) and the glucocorticoid receptor (PDB: 3CLD), using the Glide module within Schrödinger (Release 2020-2, Schrödinger LLC, New York, NY) [9-11]. For the androgen receptor (**Figures 4C, D**), the PDB: 2PIT structure was selected due to the 1.76Å resolution and good coverage of the ligand binding domain of the androgen receptor (251 residues). PT150 showed optimal binding to the peptide activator allosteric site on the androgen receptor but did not dock into the steroid binding site identified for dihydrotestosterone. For the glucocorticoid receptor, the PDB: 3CLD was selected due to the 2.84Å resolution, limited other available structures and good coverage of the ligand binding domain of the glucocorticoid receptor (259 residues). Similar to the analysis of the androgen receptor, PT150 preferentially bound to the co-activator peptide allosteric binding site of the glucocorticoid receptor (**Figures 4E, F**). No anchoring interactions were observed for the steroid binding pocket.

### PT150 Induces Differential Expression of GR- and AR-Regulated Genes, as Well as Genes for Immune Signaling and Inflammation, in Animals Infected With SARS-CoV-2

**Figure 5:**
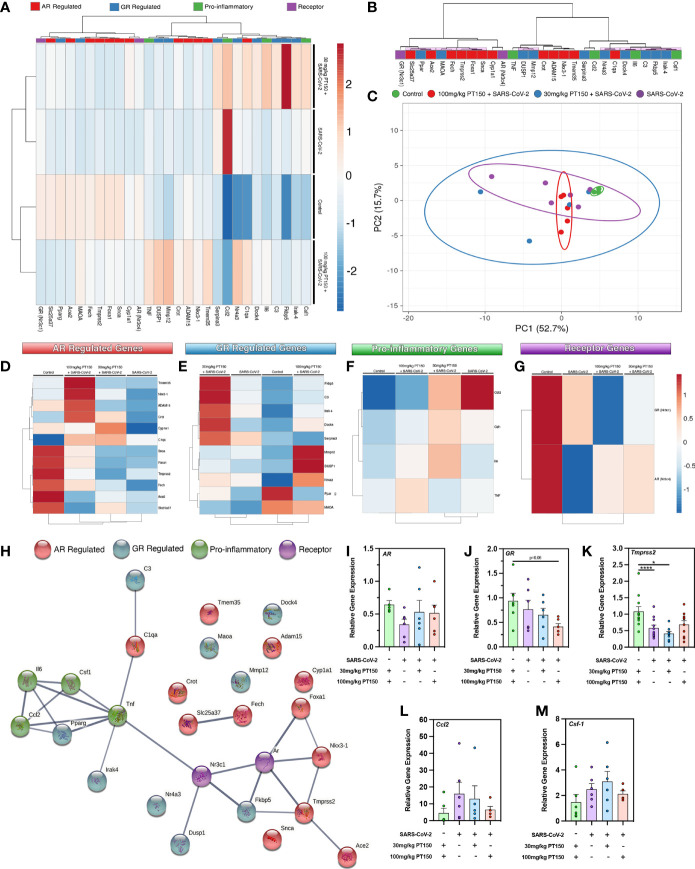
PT150 modulates the expression of AR- and GR-regulated genes in the lungs of animals infected with SARS-CoV-2. **(A)** Expression of transcripts for AR-regulated, GR-regulated, pro-inflammatory, and individual receptor genes were examined in lung tissue from hamsters using quantitative reverse transcriptase PCR. Heat maps for each gene depict ontological clustering based on expression. Clustering significance between genes based on relative gene expression is depicted by purple highlighted boxes **(B)**. Principal component analysis of all genes investigated show infection-treatment group differences (*p=0.02, **(C)**. Individual grouping heat maps of infection-treatment group analysis for AR-regulated **(D)**, GR-regulated **(E)**, pro-inflammatory **(F)** and individual receptor transcripts **(G)** during SARS-CoV-2 infection and treatment with PT150. **(H)** STRING protein-protein mapping of the functional relationships between each gene. **(I–M)** Analysis of individual gene expression demonstrates differential expression during infection with SARS-CoV-2 that is largely comparable to control levels in animals treated with 100 mg/Kg PT150. Overall expression of *GR* is decreased by PT150 **(J)**. *p<0.05, ****p<0.001; *N=6* animals per group.

To determine if treatment with PT150 directly modulates the activity of AR and GR *in vivo* in Syrian hamsters infected with SARS-CoV-2, we measured the expression of several classes of genes regulated through these receptors, as well as the expression of genes associated with inflammation and innate immune responses at 3 DPI (**Figure 5**). AR- and GR-regulated genes, inflammatory cytokines and chemokines, and respective AR and GR transcripts were assessed through RT-qPCR, where individual sample clustering is represented through heat mapping (**Figure 5A**). Statistical significance (*p<0.05) between clustering pairs is visualized by purple boxes outlining groupings generated (**Figure 5B**). Average branching analysis showed GR response elements closely associated with pro-inflammatory gene transcription, whereas AR-modulated genes clustered similarly to receptor transcripts themselves. Pearson correlation coefficient analysis of gene-gene comparison based on relative expression is shown in **Supplemental Figure 1**. Principal component (PC) analysis of all genes investigated yielded infection-treatment group differences (*p<0.05, **Figure 5C**). Individual receptor modulated gene profiles were investigated for AR (**Figure 5** **D**), GR (**Figure 5E**), pro-inflammatory (**Figure 5F**), and for individual receptors (**Figure 5G**). AR-regulated gene transcripts revealed average clustering profile similarities between 30 mg/Kg PT150+SARS-CoV-2 and SARS-CoV-2 infected animals that were dissimilar from the 100 mg/Kg PT150 + SARS-CoV-2 and control animals (**Figure 5D**). The GR-regulated gene transcripts clustered such that 100 mg/kg PT150-treated animals resembled transcript levels of control animals, where 30 mg/kg PT150 animals’ transcript levels clustered more so with the SARS-CoV-2 infected animals (**Figure 5E**). Analysis of pro-inflammatory genes revealed similar associations where low dose PT150 clustered with SARS-CoV-2 infected animals, whereas animals treated with 100mg/Kg PT150 had expression patterns of AR- and GR-regulated genes similar to controls. Individual receptor transcript level analysis revealed decreases in AR and GR transcription with PT150 treated animals, interestingly showing more prominent decreases in *GR*. STRING protein-protein interaction mapping based on the interconnection between protein functionality revealed complex associations with the genes of interest, where high confidence interactions exist between pro-inflammatory genes and GR, whereas AR predominantly regulated genes associated with viral entry such as *Tmprss2* and *Ace2* (**Figure 5H**). Individual gene transcript analysis revealed treatment group differences where SARS-CoV-2 infection modulates *AR*, *Tmprss2*, and *Ccl2* (**Figures 5I, K, L**); 100 mg/kg PT150 treatment decreased *GR* levels overall and trends were observed indicating that expression of *AR*, *Ccl2*, and *Csf-1* and *Tmprss2* were comparable with the control group in infected animals treated with 100 mg/Kg PT150 (**Figures 5J**–**M**). All other genes of interest were interrogated for differences between groups, with *C3*, *Fkbp5*, *Irak-4*, *Mmp12*, *Maoa*, *Nr4a3*, *Serpina3*, *Adam15*, *C1qa*, *Fech*, *Foxa1*, *Tmem35* and *Ace2* all found to have statistically significant difference amongst group means (**Supplemental Table 2**).

### ACE2 and TMPRSS2 Production and Regulation in Animals Infected With SARS-CoV-2

**Figure 6:**
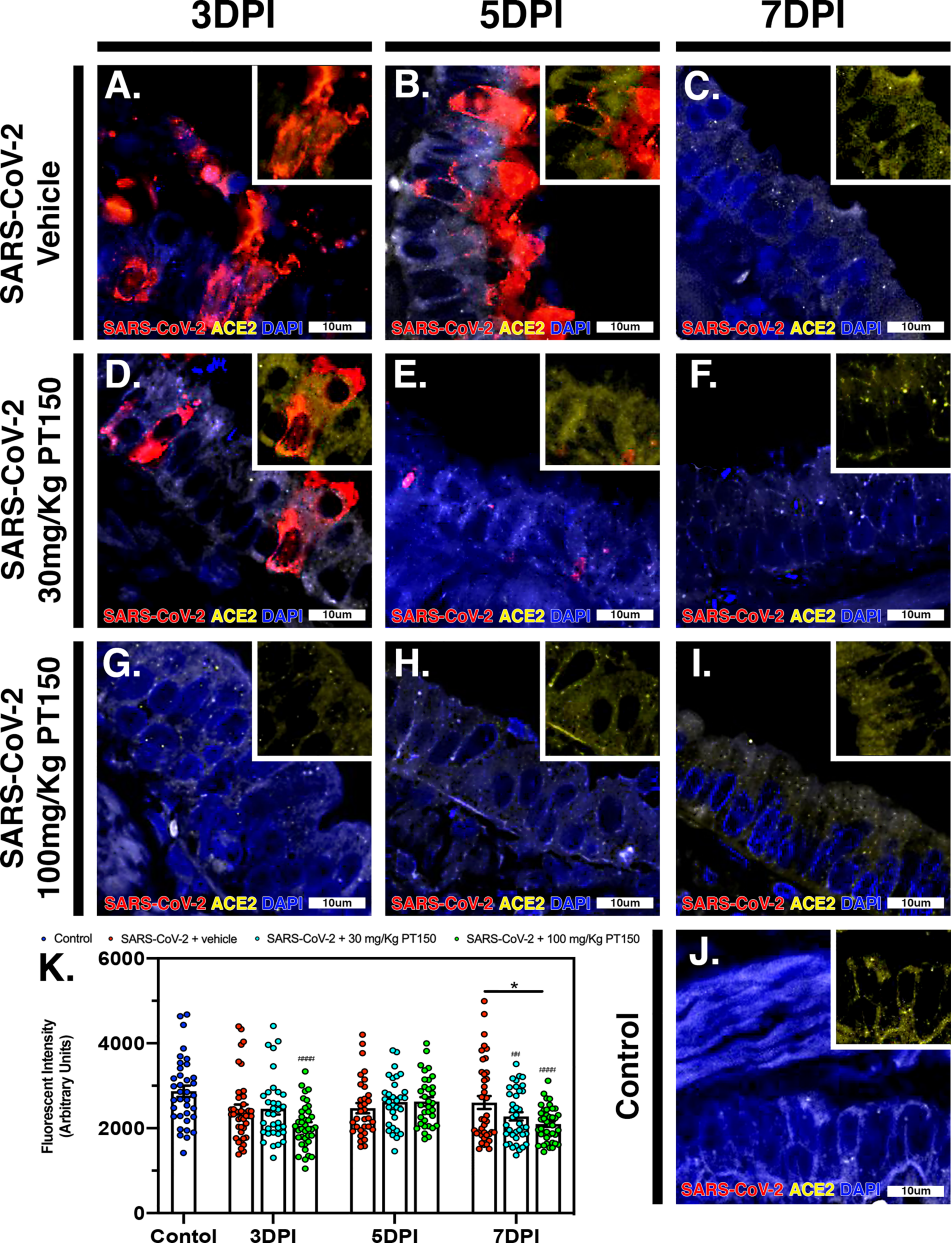
PT150 treatment decreases expression of the ACE2 receptor in lung. In Syrian golden hamsters infected with SARS-CoV-2, there was a modest decrease in expression of the ACE2 in bronchiolar epithelial cells at day 7 post-infection in animals given PT150 at 100 mg/Kg/day. Experimental groups were **(A–C)** SARS-CoV-2 + vehicle, **(D–F)**, SARS-CoV-2 + 30 mg/Kg/day PT150, **(G–I)** SARS-CoV-2 + 100 mg/Kg/day PT150, **(J)** Control + vehicle. **(K)**, Quantification of ACE2 expression in lung tissue. *p<0.05**. ^##^***p*<0.002, ^####^*p*<0.0001 compared to control; *N=6* animals per group.

**Figure 7:**
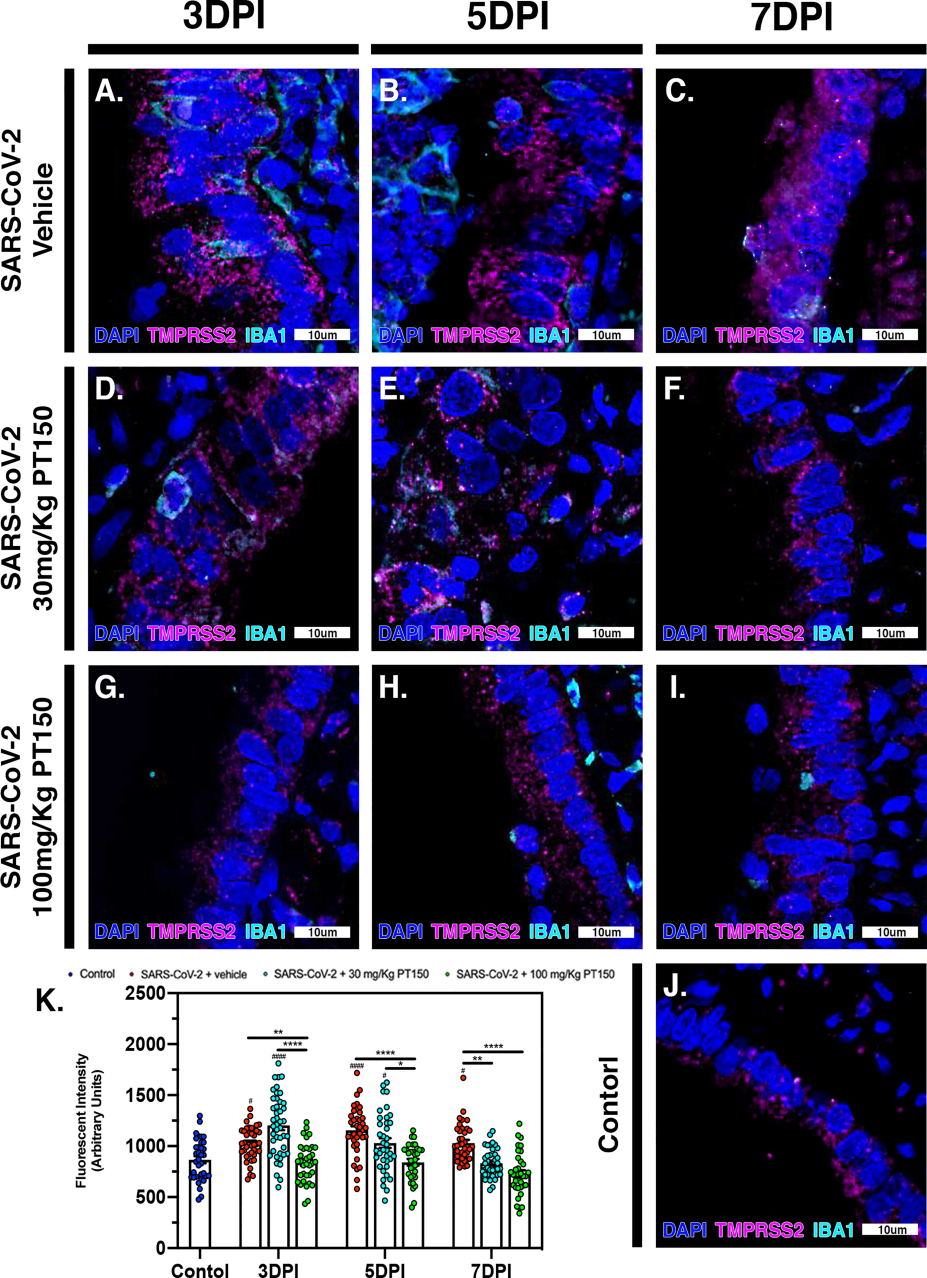
TMPRSS2 protein levels in bronchiolar epithelial cells are decreased by treatment with PT150 in animals infected with SARS-CoV-2. In Syrian golden hamsters infected with SARS-CoV-2, there was a significant decrease in levels of TMPRSS2 protein in bronchiolar epithelial cells at all timepoints in animals given PT150 at 30 and 100 mg/Kg/day. Experimental groups were **(A–C)** SARS-CoV-2 + vehicle, **(D–F)**, SARS-CoV-2 + 30 mg/Kg/day PT150, **(G–I)** SARS-CoV-2 + 100 mg/Kg/day PT150, **(J)** Control + vehicle. **(K)** Quantification of TMPRSS2 protein expression in lung tissue. ^#^*p*<0.05, ^####^*p*<0.0001 compared to control. *p<0.05, **p<0.01, ****p<0.0001; *N=6* animals per group.

The cell surface TMPRSS2 serine protease and the ACE2 receptor are required for S-protein priming and viral binding and to facilitate entry of SARS-CoV-2 into cells. We therefore examined production of these proteins in lung tissue from infected hamsters with and without PT150 treatment. ACE2 intensity measurements were quantified by immunofluorescence imaging (**Figure 6**), where pseudostratified columnar epithelium lining bronchioles showed marked decreases in levels of ACE2 relative to vehicle control (**Figure 6J**) with the administration of 100 mg/kg/day of PT150 at the 3-day timepoint as well as the 7-day timepoint (**Figures 6G**–**I, K**). Decreased ACE2 was also observed in the 30 mg/kg/day PT150 lung sections at 7 DPI (**Figure 6F**). Levels of ACE2 in vehicle-treated SARS-CoV-2 lung sections were similar to control until the 7-day timepoint, at which point production reached a maximum (**Figures 6A**–**C**). Expression of TMPRSS2 within bronchiolar cells was increased in the untreated lung sections infected with SARS-CoV-2 (**Figures 7A**–**C, K**), indicating induction associated with enhanced levels of viral entry, replication and dissemination. In contrast, the 100 mg/kg/day PT150 treated animals showed significant decreases in TMPRSS2 protein levels at all timepoints (**Figures 7G**–**I**) similar to levels observed in the control group (**Figures 7J, K**). There was also reduction in TMPRSS2 proteins levels within the 30 mg/kg/day treatment group at 5 DPI and 7 DPI (**Figures 7D**–**F, K**), similar to levels in control animals at 7 DPI.

### Inflammatory Activation of Macrophages and Release of Interleukin-6 (IL-6) Is Decreased by PT150 Treatment in Syrian Hamsters Infected With SARS-CoV-2 in Parallel With Decreased Viral Load

**Figure 8:**
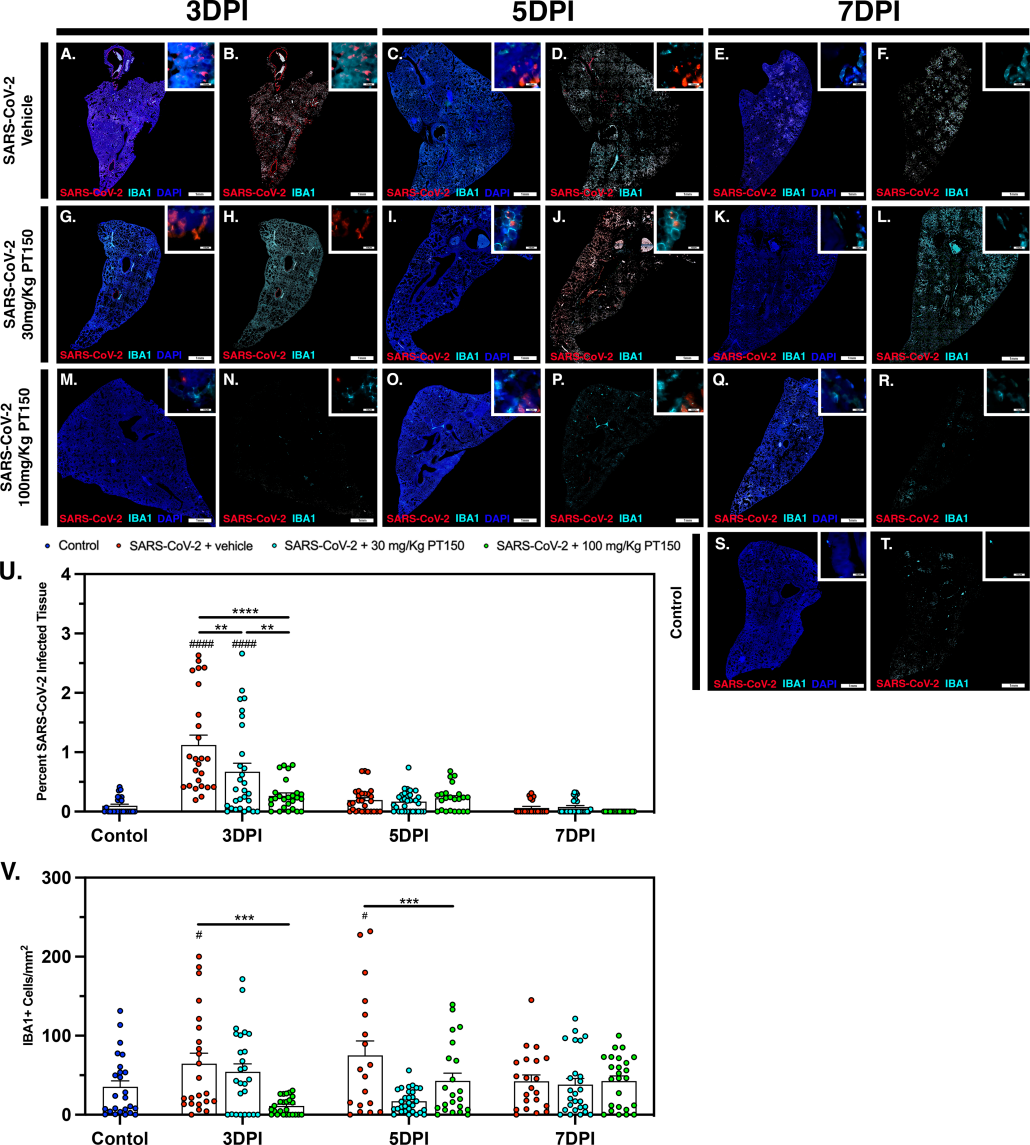
SARS-CoV-2 viral protein expression and macrophage infiltration is reduced by oral administration of PT150. Viral load was determined by immunofluorescence staining of the SARS-CoV-2 nucleocapsid protein. Total viral replication and immune cell infiltration is seen within lung sections at the 3 DPI, 5 DPI, and 7 DPI timepoints. **(A–F)**, SARS-CoV-2+Vehicle; **(G–L)** SARS-CoV-2+30 mg/Kg/day PT150; **(M–R)**, SARS-CoV-2+100 mg/Kg/day PT150; **(S, T)**, Control + Vehicle. Quantification of percent SARS-CoV-2 infected tissue **(U)** and infiltrating macrophages **(V)** was performed using adaptive intensity thresholding and cellular co-localization of protein expression. *N=6* animals per group with a sampling of 5 lobes of tissue per animal. Differences were determined by one-way ANOVA, **p<0.002, ***p<0.0002, ****p<0.0001. ^#^*p*<0.05, ^####^*p*<0.0001 compared to control.

The peak of infectivity and viral replication within the Syrian hamster model at sampled time points was observed at 3 DPI. Using quantitative immunofluorescence scanning microscopy, we evaluated the extent of lung area containing both SARS-CoV-2 viral protein and infiltrating IBA1+ macrophages (**Figure 8**). In the SARS-CoV-2 + vehicle group, staining for viral nucleocapsid protein indicated the peak of viral protein production at 3 DPI, which declined at both 5 and 7 DPI (**Figures 8A**–**E**). Increased infiltration of macrophages was present at 3DPI, peaked at 5 DPI and was then followed by a decline at 7 DPI (**Figures 8A**–**E**). Control animals (**Figures 8** **S, T**) showed no staining for SARS-CoV-2 and only background levels of IBA1. Viral replication, as measured by cellular expression of SARS-CoV-2 nucleocapsid protein, was decreased by administration of 30 mg/kg/day of PT150 (**Figures 8G**–**L**), and was further decreased with 100 mg/kg/day of PT150 (**Figures 8M**–**R**). In parallel to the decrease in viral nucleocapsid protein, there was a decrease in the percent of total lung area occupied by infiltrating macrophages at 30 and 100 mg/Kg/day PT150 (**Figures 8G**–**R**). Quantification of immunofluorescence staining, as measured by the percent of total sampled lung area positive for SARS-CoV-2, revealed marked decreases in both percent lung area expressing viral replication, as well as the number of IBA1^+^ cells/mm^2^ (**Figures 8** **U, V**). This demonstrates a dose-response relationship in therapeutic efficacy of PT150 for reducing the viral burden of SARS-CoV-2 in lung, as well as a corresponding decrease in the extent of infiltrating macrophages.

**Figure 9:**
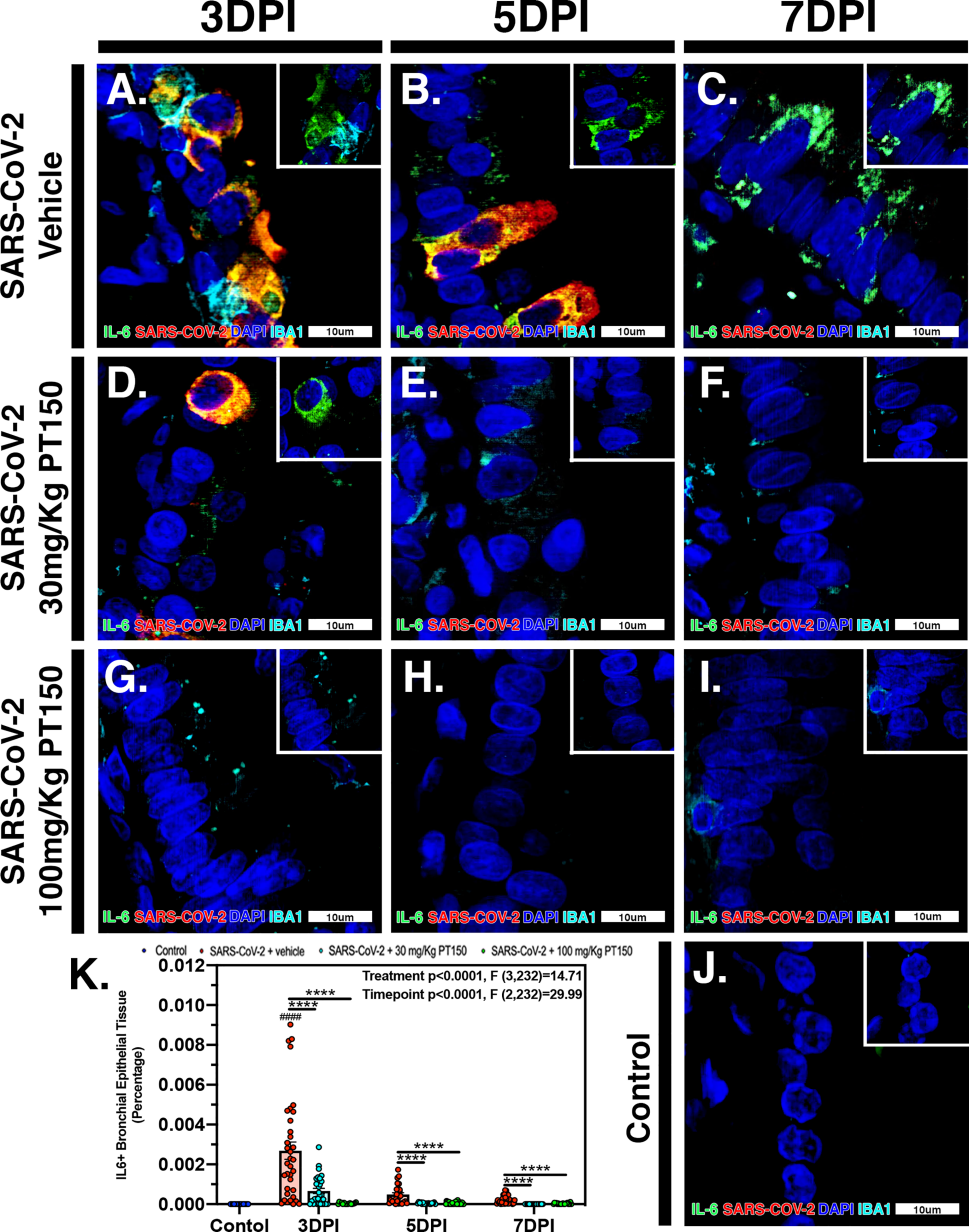
PT150 treatment decreases expression of the inflammatory cytokine Interleukin-6 (IL-6) in lung following infection with SARS-CoV-2. There was a significant decrease in expression of TMPRSS2 in bronchiolar epithelial cells of Syrian golden hamsters infected with SARS-CoV-2 at all timepoints in animals given PT150 at 30 and 100 mg/Kg/day. Experimental groups were **(A–C)** SARS-CoV-2 + vehicle, **(D–F)**, SARS-CoV-2 + 30 mg/Kg/day PT150, **(G–I)** SARS-CoV-2 + 100 mg/Kg/day PT150, **(J)** Control + vehicle. **(K)** Quantification of IL-6 protein expression in lung tissue. *p<0.05, **p<0.01, ****p<0.0001; ^####^*p*<0.0001 compared to control. *N=6* animals per group.

In Syrian golden hamsters infected with SARS-CoV-2, there was a significant increase in macrophage-derived IL-6 within the bronchiolar epithelial layer (**Figures 9A**–**C**). Immunofluorescence images of infected hamster lung tissue at 3 DPI revealed cells within the bronchiolar epithelial layer co-producing high levels of IL-6 (green) with SARS-CoV-2 nucleocapsid protein (red). Nuclei were counterstained with DAPI (blue) and IBA1+ macrophages are shown in cyan. In infected animals, triple label immunofluorescence images show cells staining intensely for IL-6 and co-localizing with expression of SARS-CoV-2 nucleocapsids protein, adjacent to IBA1^+^ macrophages (**Figure 9A** and inset) when compared with control animals (**Figure 9J**). Expression of IL-6 persisted at 5 and 7 DPI, even after SARS-CoV-2 nucleocapsid protein was no longer evident (**Figures 9B, C**). Treatment with PT150 and 30 mg/Kg/day (**Figures 9D**–**F**) and 100 mg/Kg/day (**Figures 9G**–**I**) dramatically decreased expression of IL-6, concordant with decreases in SARS-CoV-2 and in the presence of IBA1^+^ macrophages. Quantification of fluorescence data indicated differences between PT150-treated groups and infected + vehicle groups at all timepoints (Treatment, *p*<0.0001, F(3,232)=14.71; Timepoint, *p*<0.0001, F(2,232)=29.99), with the greatest differences evident at 3 DPI, where both PT150-treated groups were different from the SARS-CoV-2 + vehicle group, as well as from each other, indicating dose-dependent effects on reduction of IL-6 in the bronchiolar epithelial layer of infected hamsters (**Figure 9K**).

### PT150 Reduces CD4^+^ T-Cell Accumulation Surrounding Bronchi

**Figure 10:**
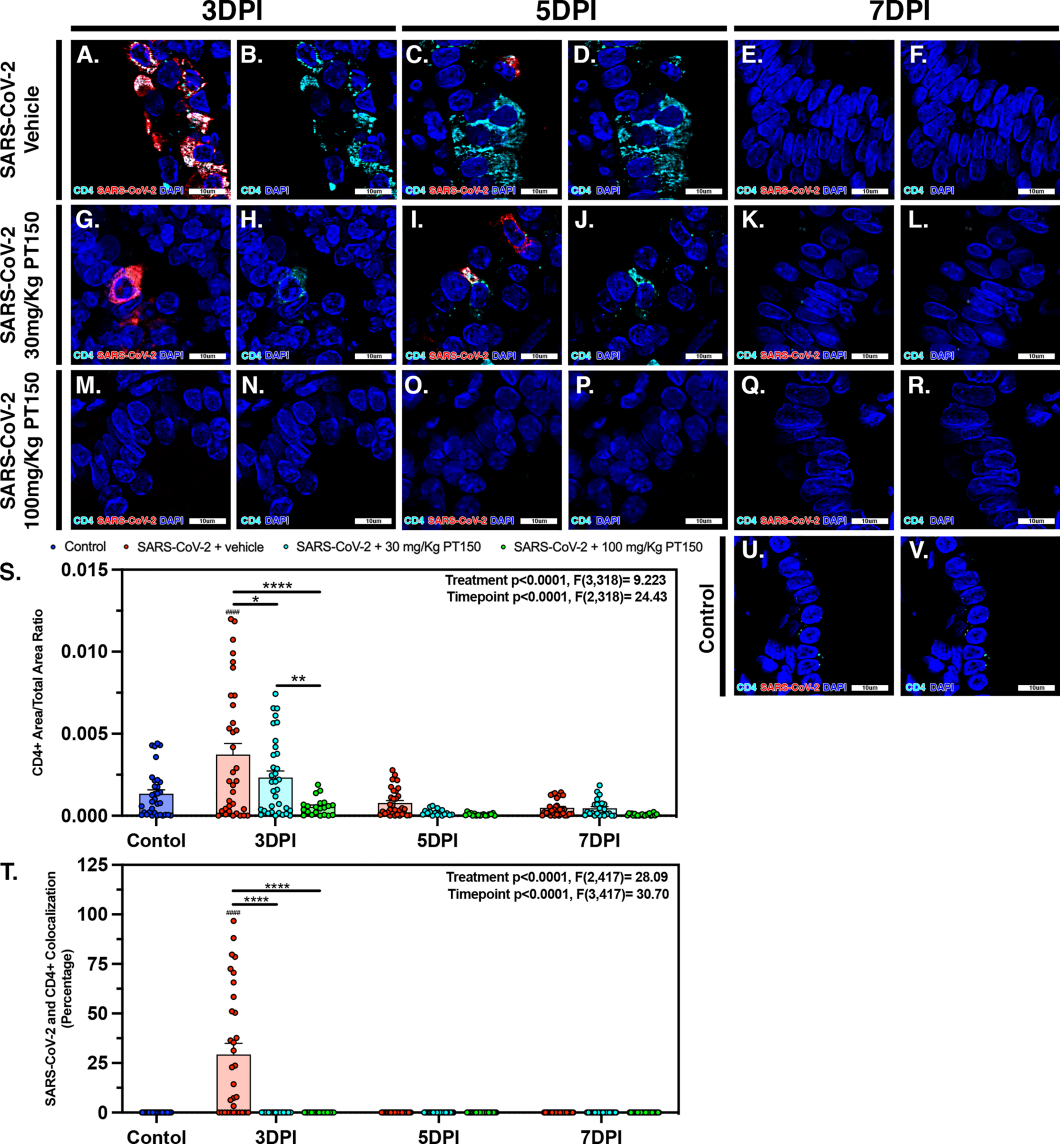
SARS-CoV-2 infected CD4^+^ T-cell populations decrease in a dose dependent manor with PT150 administration. Increases in CD4^+^ T-cell populations were observed at 3 DPI within SARS-CoV-2 infected groups **(A, B, S)**, of which was decreased in a dose-dependent manor with PT150 administration **(G, H, M, N, S)** when compared to negative controls **(U, V)**. These populations further decreased in linear fashion to 7 DPI **(C–F, I–L, O–R, S)**. CD4^+^ cells had high colocalization with SARS-CoV-2 viral reactivity, again decreasing with PT150 dosage and progression of time **(A, G, M, T)**. *p<0.05, **p<0.01, ****p<0.0001. ####p<0.0001 compared to control; *N=6* animals per group.

CD4^+^ T-cells surrounding main stem bronchi and bronchioles peaked at 3 DPI in SARS-CoV-2-infected animals and showed high intracellular levels of SARS-CoV-2, based on fluorescence co-localization (**Figures 10A, B**). T-cell populations were observed decreasing linearly until 7 DPI within this group of animals (**Figures 10C**–**F**). Treatment with PT150 decreased the total number of CD4^+^ T-cells initially observed within the epithelial margin at 3 DPI in a dose-dependent manor (**Figures 10H, N**) as well as overall levels of SARS-CoV-2 (**Figures 10G, M**). The number of CD4^+^ cells further decreased in all groups by 7 DPI (**Figures 10I**–**L**, **O**–**R**), similar to negative control animals (**Figures 10** **U, V**). Quantification of the total lung area occupied by CD4^+^ cells revealed resident CD4^+^ cell populations that increased with SARS-CoV-2 infection at 3 DPI but were greatly by treatment with PT150 (**Figure 10** **S**). The number of CD4^+^ cells co-localizing with SARS-CoV-2 was similarly decreased in the PT150-treated groups at 3 DPI, where a high percentage of CD4^+^ cells contained SARS-CoV-2 in infected animals that received vehicle (**Figure 10** **T**).

### Modeling the Temporal Sequence of Innate Immune Inflammatory Responses in Animals to Infection With SARS-CoV-2 Reveals That PT150 Mitigates the Severity of Pathological Outcomes

**Figure 11:**
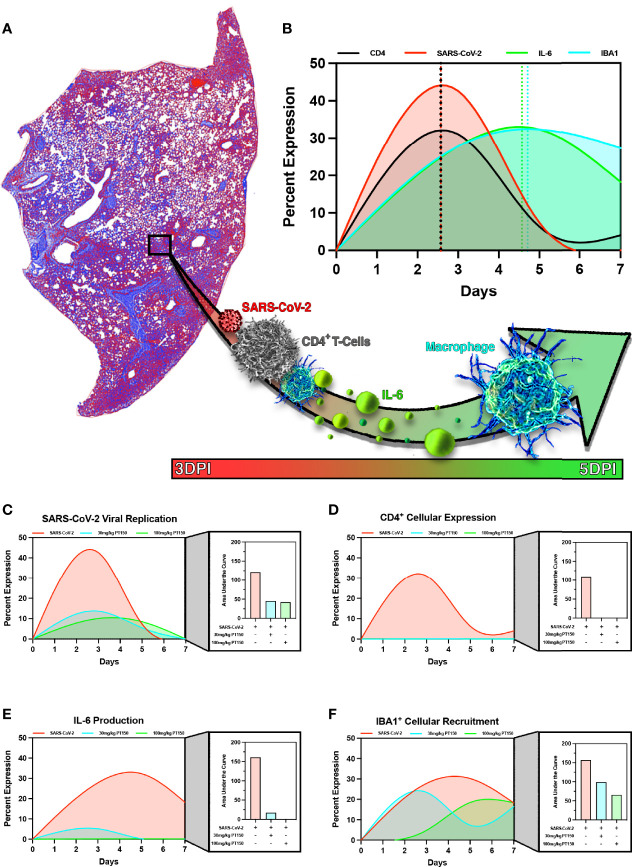
PT150 reduces the severity of immune responses and lung pathology in animals infected with SARS-CoV-2. Modeling of SARS-CoV-2 driven pathological changes in lung tissue **(A)** shows concurrent increases in SARS-CoV-2 viral nucleocapsid protein intensity and CD4^+^ populations early in the course of infection (3 DPI) followed by increases in IL-6 production and IBA1^+^ cell populations at 5 DPI **(B)**. Therapeutic treatment utilizing PT150 decreased the severity of all pathologic responses measured, based on the area under the curve (AUC) for SARS-CoV-2 **(C)**, CD4^+^ cells **(D)**, IL-6 **(E)**, and IBA1^+^ cells **(F)**.

In order to model the overall impact of treatment with PT150 on the progression of pathology observed in the lungs of hamsters infected with SARS-CoV-2, we calculated normalized pathological overlays of each cellular and molecular response measured over the 7-day study (**Figure 11**). This is schematically depicted in **Figure 11A**, demonstrating that infection with SARS-CoV-2 induced rapid increases in the number of CD4^+^ T-cells, which activate resident macrophages to produce IL-6. This increase in IL-6 recruits monocytes to areas of damage increasing overall macrophage populations leading to severe inflammatory infiltration and consolidation of lung injury. Normalized overlays modeling the temporal sequence of each cellular response (**Figure 11B**) indicated that the peak of CD4^+^ T-cell infiltration occurred simultaneously with the peak of SARS-CoV-2 infection at Day 2.5, followed by maximal expression of IL-6 at Day 4.5 that slightly preceded maximal infiltration of IBA1^+^ macrophages. Integrating each response over time reveled that PT150 treatment caused dose-dependent decreases in SARS-CoV-2 replication (**Figure 11C**), CD4+ T-cell infiltration and IL-6 production (**Figures 11D** **, E**), as well as decreased infiltration of IBA1+ macrophages (**Figure 11F**). Notably, the peak of macrophage infiltration was significantly delayed at the higher dose of PT150 (100 mg/Kg). Modeling of these cellular responses demonstrates that PT150 positively modulated the progression of lung pathology during infection with SARS-CoV-2.

## Discussion

Treatment options available for individuals infected with SARS-CoV-2 continue to be limited. Of the few treatments that are available, many are the result of repurposing efforts and have low efficacy and limited experimental characterization surrounding the direct mechanisms of action against SARS-CoV-2. Therapeutic approaches have primarily focused on targeting viral proteins (43–45) or the host immune response through corticosteroid administration, which can be detrimental if not administered correctly during the course of infection (46). However, less research has been published on molecules that have both anti-viral activity as well as immunomodulatory activity to decrease the hyperinflammatory response to SARS-CoV-2 infection. The mechanism of action of PT150 involves modulation of GR and AR that inhibits viral entry and replication by decreasing expression of two main proteins used by SARS-CoV-2 for endosomal uptake, TMPRSS2 and ACE2 (9, 47–52). The beneficial effects of PT150 observed on inflammatory immune responses to SARS-CoV-2 also likely involve its effects on GR, which is expressed in numerous immune cells including macrophages, T cells, dendritic cells and epithelial cells (53, 54). PT150 was administered orally to animals infected with SARS-CoV-2, resulting in marked clinical and pathological improvements when compared to the infected vehicle control group. Because Syrian hamsters can propagate human SARS-CoV-2 and show a high degree of sequence homology in both AR and GR with the human receptors (**Figure 4**), it is reasonable to surmise that SARS-CoV-2 infected patients will response similarly to PT150 treatment.

Clinical signs of disease in SARS-CoV-2-infected hamsters, such as akinesia and 5-10% maximal weight loss was similar to features reported in other COVID-19 studies in Syrian hamsters (55–61). Animals that received PT150 at clinically relevant low (30 mg/kg/day) and high (100 mg/Kg/day) doses did not display as severe clinical manifestations and weight loss and showed an overall reduction in viral titer and genome copies (**Figure 1**), as well as a marked reduction in inflammatory cellular infiltration and bronchiointerstital pneumonia (**Figure 2**). These clinical and histopathological findings were mirrored by the reduction in lung hypercellularity, as indicated by digital image analysis of whole-lung scans in the infected vehicle- and PT150-treated animals (**Figure 3**). Lung hypercellularity in infected animals increased throughout the course of the 7-day study, similar to the progressive pneumonia experienced by SARS-CoV-2 patients in response to the overwhelming amount of edema and cellular infiltration into the parenchyma of the lungs. Treatment with PT150 markedly reduced hypercellularity at 5 and 7 DPI, with the low-dose PT150 group showing reduction at 7 DPI. This indicates that PT150 may be regulating cellular infiltration and recruitment into the lung tissue, resulting in reduced pathology and inflammation.

We performed phylogenetic analysis and molecular docking studies to characterize the putative molecular targets by which PT150 could modulate disease progression (**Figure 4**). Sequence concordance values for both AR and GR were very high across species representative of divergent taxonomic groups, including the greater horseshoe bat, sunda pangolin, Syrian golden hamster and human (amongst other species). This underscores not only the likely similar patterns of regulation of AR/GR-dependent genes across species that are required for entry of SARS-CoV-2, such as ACE2 and TMPRSS2, but also the predictive potential for therapeutic modulation of these receptors using PT150, given the sequence similarity between the hamster and human genes. The similarity in these regulatory proteins also highlights the potential for cross-species transmission of SARS-CoV-2 and related beta-coronaviruses and the importance of identifying compounds that can modulate signaling of AR and GR to increase host defense.

Molecular docking studies (**Figures 4C, D**) indicated likely interactions with the co-activator site of the ligand binding domain (LBD) of both receptors (shaded in green), rather than the steroid binding pocket (shaded in magenta in each structure). Thus, PT150 may function as an allosteric modulator of these receptors, resulting in transcriptional repression of key genes, such as TMRPSS2 and ACE2, as well as pro-inflammatory factors. Modeling was conducted using the published crystal structures for AR (PDB: 2PIT) (25) and GR (PDB: 3CLD), solved as a complex co-crystalized with flutacasone (26). PT150 did not favorably bind to the steroid site of the human LBD in any simulation and although the binding site has some flexibility in both receptors, the stereochemistry of PT150 appears to hinder binding at this site. The co-activator peptide binding site most favorably interacted with PT150, suggesting this as a site of allosteric modulation that could inhibit transcriptional activity without directly competing for binding with endogenous corticosteroid ligands. This has been reported for small molecule modulators of other nuclear receptors, such as NR4A2/Nurr1, where molecular docking studies indicated interactions at the co-activator peptide binding site that correlated with transcriptional inhibition of inflammatory gene expression (62). Studies published with the 1NHZ structure of GR co-crystalized with RU486 suggested potential interactions within the steroid binding pocket of the LBD (63), but the helix that forms part of both the steroid site and the co-activator peptide site was not complete in the 1NHZ structure due to a 9 amino acid deletion, resulting in a distortion of the GR LBD that would be more permissive of interactions within the steroid binding pocket. Thus, PT150 may modulate both AR and GR signaling as an allosteric inhibitor through interactions with the co-activator domain, leading to decreased transcription of target genes regulated by these receptors.

Analysis of AR- and GR-regulated gene expression in hamsters infected with SARS-CoV-2 with and without treatment indicated that PT150 directly modulated the expression of numerous genes such as *Fech*, *Dock4*, *Dusp1*, *Fkbp5*, *Maoa*, *Nr4a3*, *Serpina3*, *Adam15*, *Cyp1a1*, *Slc25a37* known to be regulated through these nuclear receptors (64–66). Structurally, AR and GR are similar in that each contains a zinc-finger motif within the DNA binding domain, as well as having ligand binding domains that, when activated, recruit nuclear chaperone proteins that promote nuclear localization and subsequent transcriptional activation (67). Activation of glucocorticoid response elements (GSEs) and androgen response elements (AREs) in the 5’-flanking regions of *Tmprss2, Ace2, Il6, Tnf, and Il1b* have been shown to be regulated through AR, GR or both (68, 69). Modulation of inflammatory gene expression (**Figure 5H**) suggests PT150 promotes interactions between GR and the NF-κB-regulated genes *Csf1*, *Il6*, *C1qa*, and *C3* (64, 70–73). Decreased expression of *Csf1* and *Il6* in PT150-treated animals is consistent with the observed decrease in infiltrating macrophages (**Figure 3**) and suggests a stronger associated between GR and regulation of pro-inflammatory genes. In contrast, interactions between PT150 and AR seem to associate more closely with genes associated with viral entry, such as *Tmprss2*, *Ace2*, *Foxa1* and *Fkbp5*. These findings highlight the importance of these corticosteroid receptors for modulating both viral entry and immunologic response to infection with SARS-CoV-2 and utility of PT150 in modulating their activity. Analysis of differential expression patterns of AR- and GR-regulated genes in animals infected with SARS-CoV-2 indicated that treatment with PT150 reduced expression of *Tmprss2*, *Il-6*, *Csf-1*, *Ccl2* and *GR* transcript levels in the lungs of infected animals. Determination of ontological clustering by clade and by PC analysis across all genes investigated indicated that transcriptional changes in GR-regulated genes were greater than those observed for AR-regulated genes (**Figure 5**). These findings are consistent with genome-wide ChIP-Seq studies that identified AR binding sites in *cis*-acting consensus sequences in the promoters of *Tmprss2* and *Ace2*, as well as putative GR binding sites in the promoters of each gene (66). These studies and others (9, 65) indicate that regulation of TMPRSS2 and ACE2 by androgens and glucocorticoids may enhance susceptibility to infection with SARS-CoV-2, suggesting that approaches that downregulate gene expression dependent on these receptors could mechanistically decrease viral entry by modulating host defense. The observed decrease in transcript levels of both AR- and GR-regulated genes in lungs of infected hamsters treated with 100 mg/Kg PT150 supports the conclusion that this compound is a direct modulator of AR and GR *in vivo*, as suggested by the *in silico* molecular docking data in **Figure 4**.

To further investigate the molecular targets of PT150 *in vivo*, expression of ACE2 was determined in lung in bronchiolar epithelial cells (**Figure 5**). Co-localization of SARS-CoV-2 and ACE2 were observed in bronchiolar epithelial cells at 3 DPI and 5 DPI, supporting that ACE2 is a vital component in viral binding and entry (**Figures 5A**–**C**). This demonstrates that viral infectivity may transcriptionally regulate ACE2 production to promote viral replication. Treatment with 100 mg/Kg PT150 decreased the overall amount of ACE2 in addition to reducing co-localization with SARS-CoV-2. This reduction of ACE2 could be due to inhibition of AR binding to the promoter of *Ace2*, thereby decreasing transcriptional activation, as reported for other anti-androgens (74). Expression of TMPRSS2 was also investigated due to the requirement of this cell surface serine protease for processing of the viral S1 spike protein that is necessary for viral entry in complex with ACE2 (**Figure 6**). Protein levels of TMPRSS2 in infected animals treated with vehicle increased at all time points, demonstrating an association with viral replication. The low-dose PT150 treatment (30 mg/Kg) decreased TMPRSS2 expression at 5 and 7 DPI, whereas treatment with 100 mg/Kg decreased TMPRSS2 expression at all timepoints, demonstrating a dose-response effect in modulating expression of TMPRSS2 in lung. Decreased production of both TMPRSS2 and ACE2, as well as the corresponding decreases in mRNA in PT150-treated animals (**Figure 5**), supports that PT150 is a negative transcriptional regulator of these genes (74, 75). These data suggest that the anti-viral activity of PT150 is due to direct modulation of host defense that decreases viral entry points.

To determine if PT150 was effective at reducing overall viral replication, infection and immune responses, whole lung sections were analyzed, and viral infectivity was determined by assessment of the SARS-CoV-2 nucleocapsid protein (**Figure 7**). In untreated animals, there was a significant change from control in the percentage of lung area infected with SARS-CoV-2 at 3 DPI that decreased by 5 DPI and 7 DPI, as seen in previously published studies (57, 76, 77). Viral loads in animals administered PT150 at 100 mg/Kg were not different from control at 3 DPI, demonstrating a dramatic reduction in viral attachment, replication and dissemination throughout lung tissue, both in the bronchi and in the parenchyma. Macrophage infiltration per area of tissue in untreated animals was increased at 3 DPI and peaked at 5 DPI, with resolution occurring at the 7-day timepoint. Animals treated with the high dose of PT150 showed significant decreases in invading macrophage populations at the 3-day timepoint and the low-dose PT150 group showed decreases at 5 DPI, demonstrating a marked reduction in the severity of inflammatory infiltration of macrophages (**Figure 8**).

IL-6 is produced by alveolar type II (ATII) cells and activated macrophages, which is known to be a critical mediator of lung injury in COVID-19 patients (78). This cytokine is responsible for recruitment and activation of resident immune cells and recruitment of circulating inflammatory cells, which is closely associated with the hyperimmune response observed in patients. This response is characterized by severe infiltration of macrophages and lymphocytes into lung tissue followed by subsequent fibrosis and restriction of the airways. The presence of resident CD4^+^ T-cells was investigated for the role in activation and progression of inflammation in response to infection with SARS-CoV-2 (**Figure 10**). These cells show peak population densities at 3 DPI along that co-localized with SARS-CoV-2 virus. AT-II cells are capable of producing IL-6 upon antigen insult that is recognized by CD4^+^ cells. These cells then produce CD-21, known for polarizing alveolar macrophages from M2 resting states to M1 activated states and inducing significant increases in IL-6 production that causes fibrotic lesioning and ultimately loss of physiological function (79, 80). This activation pattern is consistent with the findings presented here, where infected animals treated with vehicle showed peak IL-6 production at the 5 DPI timepoint in association with the increases in macrophage populations in the lung, decreasing steadily by the 7-day timepoint (**Figure 9**). Interestingly, both the low- and high-dose treatment groups showed marked reduction in IL-6 levels at all timepoints. This highlights the efficacy of PT150 in reducing early-stage immune responses concomitant to mitigating the severity of progressive lung pathology in response to SARS-CoV-2 infection.

In conclusion, these data demonstrate that the progression of pathology within the lungs of SARS-CoV-2-infected animals depends upon production of both ACE2 and TMPRSS2, which facilitate viral entry and replication, leading to CD4^+^ T-cell proliferation and further recruitment of circulating macrophages that initiate a severe innate immune response leading to broncho-interstitial pneumonia and consolidation of the lung parenchyma. Expression of IL-6 is necessary for recruitment of immune cells to the site of infection, which leads to the ‘cytokine storm’ observed in patients that is associated with poorer outcomes (81, 82). PT150 treatment interrupts this progression of disease by limiting viral entry, thereby reducing viral loads and decreasing the severity of the immune response to SARS-CoV-2 infection (**Figure 11**). This may occur through allosteric inhibition of AR and GR, which decreases transcriptional activation and resulting protein levels of ACE2 and TMPRSS2, as well as mitigating excessive immune responses through inhibition of inflammatory gene expression. Importantly, decreased production of IL-6 by resident immune cells within the lung tissue is likely to correlate with an improve prognosis for patients. The novel mechanism of action of PT150 as both an inhibitor of viral entry and an immunomodulator makes it a strong candidate for evaluation as a therapeutic intervention in the treatment of COVID-19, independent of the increasing number of SARS-CoV-2 variants.

## Data Availability Statement

The original contributions presented in the study are included in the article/**Supplementary Material**. Further inquiries can be directed to the corresponding authors.

## Ethics Statement

The animal study was reviewed and approved by Institutional Animal Care and Use Committee, Colorado State University.

## Author Contributions

SR, RT, and RS performed initial conceptualization of experimentation. SR, AF, and RT conceived original project design. SR, AF, AL, JC, PR, CM, and DA performed experimentation. SR, AL, JC, TA, PR, and DA performed formal data curation and subsequent analysis. SR wrote original draft. AF, AL, JC, TA, PR, CM, DA, KP, RK, TS, NT, RS, and RT edited subsequent draft versions. RT obtained funding for this research, provided resources and supervision. All authors contributed to the article and approved the submitted version.

## Funding

This work was supported by National Institutes of Health grant ES021656 (RBT), National Institute of Allergy and Infectious Disease R01 AI140442 (TS), and the National Science Foundation 2033260 (TS).

## Conflict of Interest

The authors declare that the research was conducted in the absence of any commercial or financial relationships that could be construed as a potential conflict of interest.

## Publisher’s Note

All claims expressed in this article are solely those of the authors and do not necessarily represent those of their affiliated organizations, or those of the publisher, the editors and the reviewers. Any product that may be evaluated in this article, or claim that may be made by its manufacturer, is not guaranteed or endorsed by the publisher.
