# Supplementary material for: A Novel Glucocorticoid and Androgen Receptor Modulator Reduces Viral Entry and Innate Immune Inflammatory Responses in the Syrian Hamster Model of SARS-CoV-2 Infection
Source: Front Immunol. 2022 Feb 16;13:811430. doi: 10.3389/fimmu.2022.811430 (PMC8889105; doi:10.3389/fimmu.2022.811430)
Supplement: Supplementary file 1 [file DataSheet_1.docx]

Supplementary Material

# Supplementary Figures and Tables

## Supplementary Table 1. Primer sequences used in qRT-PCR studies.

| Gene Name | Forward Primer 5’-3’ | Reverse Primer 5’-3’ |
| --- | --- | --- |
| *Actb* | AATTTCCCTCTCGGCTGTGG | CTGGTCGTACCACTGGCATT |
| *C3* | GGTTCAGATGGCCGAGGATG | GGCCAAACTTCTCCCACTGT |
| *Dock4* | GGGGTTTTGCTTTCCTTGCG | CCGGTGTAGCACTTCGGAAA |
| *DUSP1* | CTCGTCCAGAAGCACCACTG | CTGGAGCATATCGTGCCCAA |
| *Fkbp5* | ATCTTCTCCCTGTTCAACCACG | AATGACTACTGATGAAGGCACCA |
| *Irak-4* | TAGAGAAGCTGCAACGGTGG | GTCTGGCCTTGAGGAGTCAG |
| *Mmp12* | AGTGCCAGAGCCACACTATC | AGATGGGTCAAAGATGGCTGC |
| *MAO-A* | TTAGCTGCTCGTTCTCCAGC | TACTTTGCAGGCACGGAGAC |
| *Nr4a3* | CGAGGGCTTGAAGTGGAAGA | CTGGACCCGCAGATGAAGG |
| *Pparγ* | TGTGGAGCAGAAATGCTGGA | TGGCCCACCAACTTTGGAAT |
| *SerpinA3* | CAGGTACAGCTCAGCACAGG | GCCCTTGCCTTCTCCTTGAA |
| *ADAM15* | ACATCATTGCCCAGGTCCAG | GGACAACTACAGTGCCAGGG |
| *C1qa* | ATTTCCTGCAACCCCATCCTT | TGGCCATGACCCTGGTATCC |
| *Crot* | GGCAAACATCTCACCAAGAAGG | ATGCAAGCTGAAGTGCAAGC |
| *Cyp1a1* | GCCTTAGACCCAGCTTCTGC | GGTCAAAGTGGCCAACCTCT |

| *Fech* | GGCACCATTCATCGCCAAAC | GGACAGCTCATCCAGCAACT |
| --- | --- | --- |
| *Foxa1* | AGCCTTTCCGTGCACACC | GAAAGTCGCAAGGACCCCTC |
| *Nkx3-1* | GCCAGTTGTTTTCGCTTGGT | GCTCATCTGGCCAAGAACCT |
| *Slc25a37* | GGCAGCTGGAGAATCGAACT | CCTGGGACTTTCCCACAGAC |
| *Snca* | CAGTGGCTGCAGCAATGTTC | TGACAAATGTTGGAGGGGCA |
| *Tmem35* | TACAAGGAGGGTTGCTGCTC | TCGGTGATCCTCTCAAACGC |
| *Ace2* | TGGACTTCAGATGCTCAGGTG | CACGAGATGGGACACATCCAA |
| *Tmprss2* | CTCCAGGAATCGGGCCTTAC | GCACTGGAGGTGGGTAGTTC |
| *AR* | GAAGCCATAGAGCCAGGAGTG | TACAAGCTGTCTCTCGCCAAG |
| *GR* | TTCAGCAAGCCACTACAGGG | TTGTGGTAATGTTGCGGGGA |
| *Il-6* | TGTAACCAAACCTCCGACTTG | CACAAGTCCGGAGAGGAGAC |
| *Csf1* | CGCTTTAAAGGCAACACCCC | AGTGAAGCAACTCTTCAGTCTCA |
| *Ccl2* | GACTGGGTCCAGGCATACAC | GGAGCTTGCGTCAAGTTAGC |
| *Tnf* | CGGACAGGAGGTTGACGTTAT | TCCCAGGTTCTCTTCAGGGG |

## Supplementary Table 2. Analysis of individual gene expression between treatment groups.

| **Gene** | **Treatment Group Relative Gene Expression** | | | | **One-way ANOVA** |
| --- | --- | --- | --- | --- | --- |
|  | **Control** | **100mg/kg PT150 +SARS-CoV-2** | **30mg/kg PT150 +SARS-CoV-2** | **SARS-CoV-2** |  |
| *C3* | -0.522 | -0.217 | 0.788 | -0.049 | ** |
| *Dock4* | -0.439 | 0.104 | 0.625 | -0.290 | p=0.3314 |
| *Dusp1* | -0.592 | 1.184 | -0.151 | -0.440 | p=0.1464 |
| *Fkbp5* | -1.922 | -0.479 | 2.759 | -0.358 | *** |
| *Irak-4* | -0.923 | -0.078 | 0.926 | 0.075 | ** |
| *Mmp12* | -0.830 | 1.347 | -0.391 | -0.126 | * |
| *Maoa* | 0.594 | 0.431 | -0.421 | -0.604 | * |
| *Nr4a3* | -1.798 | 1.227 | 0.428 | 0.143 | * |
| *Ppar-gamma* | 0.705 | -0.373 | 0.060 | -0.392 | p=0.3714 |
| *Serpina3* | -0.582 | -0.792 | 0.940 | 0.433 | ** |
| *Adam15* | 0.211 | 0.665 | -0.374 | -0.502 | ** |
| *C1qa* | -1.781 | 0.866 | 0.933 | -0.018 | **** |
| *Crot* | -0.189 | 0.381 | 0.145 | -0.336 | p=0.5874 |
| *Cyp1a1* | 0.111 | 0.027 | 0.342 | -0.480 | p=0.3494 |
| *Fech* | 0.462 | 0.152 | -0.176 | -0.438 | ** |
| *Foxa1* | 0.723 | 0.155 | -0.469 | -0.409 | * |
| *Nkx3-1* | -0.053 | 0.328 | -0.033 | -0.242 | p=0.6978 |
| *Slc25a37* | 0.560 | -0.469 | 0.167 | -0.258 | p=0.4392 |
| *Snca* | 0.748 | 0.116 | -0.424 | -0.439 | p=0.3662 |
| *Tmem35* | -0.109 | 0.974 | -0.406 | -0.458 | ** |
| *Ace2* | 0.610 | -0.336 | -0.055 | -0.219 | *** |
| *Tmprss2* | 0.736 | -0.007 | -0.521 | -0.207 | ** |
| *Il6* | -0.840 | 0.244 | 1.240 | -0.643 | p=0.0849 |
| *Csf1* | -0.997 | -0.207 | 0.976 | 0.229 | p=0.2680 |
| *Ccl2* | -2.354 | -1.505 | 1.286 | 2.572 | p=0.4078 |
| *Tnf* | -0.216 | 0.833 | -0.605 | -0.012 | p=0.4978 |
| *Nr3c4* | 0.385 | 0.020 | 0.067 | -0.471 | p=0.4136 |
| *Nr3c1* | 0.522 | -0.595 | -0.086 | 0.159 | p=0.0940 |

## Supplementary Figure 1. Pearson correlation coefficient in relative expression gene-gene comparison.

##
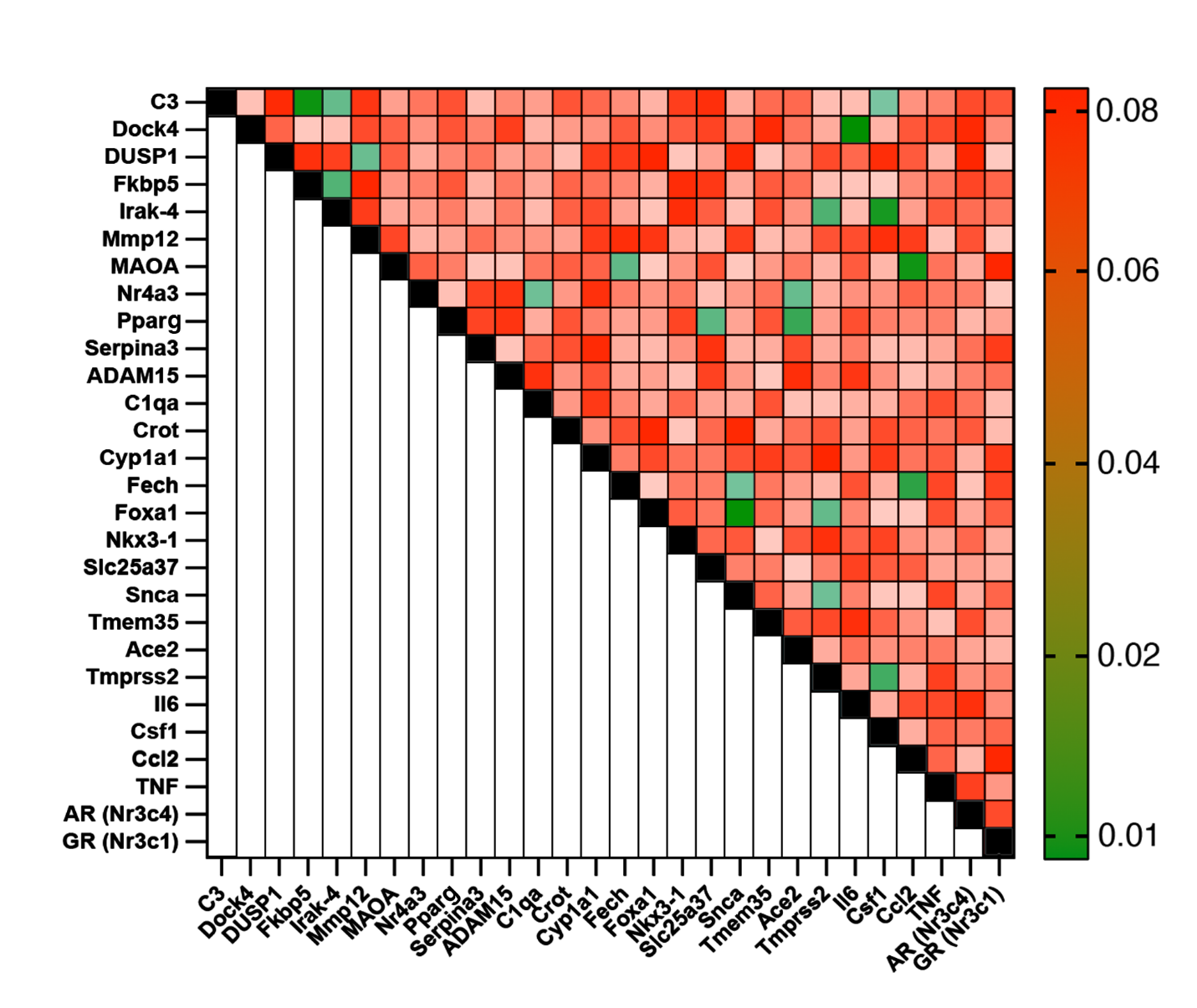


**Supplemental Figure 1.** Respective *p*-values for relative gene expression through analysis of the Pearson correlation coefficient. Significant correlations (**p*<0.05) are visualized in green, where increasing red intensity corresponds to increasing *p*-value for transcription co-expression.
